# Identification of factors influencing hydrologic model performance using a top‐down approach in a large number of U.S. catchments

**DOI:** 10.1002/hyp.13566

**Published:** 2019-11-05

**Authors:** Carolina Massmann

**Affiliations:** ^1^ Institute for Hydrology and Water Management (HyWa) University of Natural Resources and Life Sciences Vienna Austria; ^2^ Department of Civil Engineering University of Bristol Bristol UK

**Keywords:** basins, catchment hydrology, large sample hydrology, model performance evaluation, rainfall–runoff modelling, signatures, ungauged

## Abstract

Investigating the performance that can be achieved with different hydrological models across catchments with varying characteristics is a requirement for identifying an adequate model for any catchment, gauged or ungauged, just based on information about its climate and catchment properties. As parameter uncertainty increases with the number of model parameters, it is important not only to identify a model achieving good results but also to aim at the simplest model still able to provide acceptable results. The main objective of this study is to identify the climate and catchment properties determining the minimal required complexity of a hydrological model. As previous studies indicate that the required model complexity varies with the temporal scale, the study considers the performance at the daily, monthly, and annual timescales. In agreement with previous studies, the results show that catchments located in arid areas tend to be more difficult to model. They therefore require more complex models for achieving an acceptable performance. For determining which other factors influence model performance, an analysis was carried out for four catchment groups (snowy, arid, and eastern and western catchments). The results show that the baseflow and aridity indices are the most consistent predictors of model performance across catchment groups and timescales. Both properties are negatively correlated with model performance. Other relevant predictors are the fraction of snow in the annual precipitation (negative correlation with model performance), soil depth (negative correlation with model performance), and some other soil properties. It was observed that the sign of the correlation between the catchment characteristics and model performance varies between clusters in some cases, stressing the difficulties encountered in large sample analyses. Regarding the impact of the timescale, the study confirmed previous results indicating that more complex models are needed for shorter timescales.

## 1 INTRODUCTION

Hydrologic models can be developed following bottom‐up or top‐down strategies (Littlewood, Croke, Jakeman, & Sivapalan, 2003). The former relies on physically based models that describe the system of interest considering the processes observed at small spatial and temporal scales using relationships derived through observation and experimentation. As this approach has been very successful in physics (e.g., universal law of gravity), there has been much interest in applying it to other areas (Dooge, 1986). However, unlike physics, other disciplines face difficulties in conducting reproducible experiments in which the influential variables can be successfully controlled (Young, Parkinson, & Lees, 1996). This has not prevented its spread to hydrology, a field that already had high expectations for this type of models 45 years ago. It was envisioned that developments resulting from more sophisticated observational methods would lead to improved mathematical descriptions solvable with increasing computing power (Freeze & Harlan, 1969). Much work has been done in this area, and today, physically based models are widely used (Beven, 2002).

The top‐down approach, on the other hand, starts by looking at the system at a large scale. Depending on our study target, this might mean that we either (a) use a large number of entities and apply statistical techniques for inferring the properties of individual items (Dooge, 1986) or (b) analyse, for instance, a catchment at a large scale at then try to infer the processes happening at smaller temporal and spatial scales in the same catchment.

Statistical top‐down approaches do not explain how systems work; instead, they aim at extracting information about specific catchments using a large databases. Although these applications might be considered black boxes, offering little opportunity for learning about how systems function (Littlewood et al., 2003), it is recognized that this approach has a large potential when adding a postprocessing step for interpreting the results and looking for patterns (Sivapalan, Blöschl, Zhang, & Vertessy, 2003).

Applications of the top‐down approach for individual catchments often focus on identifying the required model complexity for a given application. In such cases, the analysis starts with a simple model. The modeller assesses then the performance of the model and tries to identify which missing process might be responsible for the deviations between the observed and measured hydrographs (or other system responses). This process is then added to the model, and the procedure is repeated until model performance is adequate. This top‐down approach for individual catchments was introduced into hydrology by Klemeš (1983) and has been widely used in since then (Atkinson, Woods, & Sivapalan, 2002; Bai, Wagener, & Reed, 2009; Eder, Sivapalan, & Nachtnebel, 2003; Farmer, Sivapalan, & Jothityangkoon, 2003; Montanari, Sivapalan, & Montanari, 2006; Sivapalan et al., 2003). It has helped to increase our understanding about the dominant processes relevant at different time scales and locations, as well as about the relationships between model complexity, time scale and catchment, and climate properties. Bai et al. (2009) explain that one goal of these studies is to enable the selection of an adequate model for a given catchment by looking just at the climate and catchment properties and thus not have to rely on discharge measurements. This requires that the analyses are carried out for a high number of catchments covering a large part of the observed variability, so that the relationships between catchment properties and model performance can be identified with confidence (Gupta et al., 2014). This approach has thus elements from the statistical top‐down approach (i.e., obtaining information about individual catchments on the basis of insight gained through the analysis of many catchments) and from the top‐down approach for each of these catchments (i.e., going from large to smaller scales).

This study is inspired by the work of Atkinson et al. (2002) that looked at the relationship between model complexity and timescale in nine New Zealand catchments. They inferred that more complex models are required for modelling drier catchments and for analyses at higher temporal resolutions. Because Atkinson's study considered only few catchments in a smaller area, it is useful to find out if the conclusions are also valid in other places, or if we need to identify more adequate and local predictors between model performance and catchment properties. Motivated by this question, this study is based on a systematic top down analysis with eight conceptual hydrological models of varying complexity in 574 catchments in the United States. These catchments cover a large range of climate and catchment properties and are therefore suitable for analysing the relationships between climate/catchment properties and model performance.

Although Atkinson et al. (2002) analysed only few catchments and thus were able to evaluate model performance visually, it was necessary to resort to another approach for dealing with the high number of catchments in the present study. This approach is based on the framework developed by Bai et al. (2009), which allows an automatized analysis of model performance. The main objective of this study is to identify relationships between catchment properties, model complexity, timescale, and model performance. This information might be helpful for identifying the models able to reach an acceptable performance for a given signature in a defined catchment. This is especially important in ungauged basins, where the lack of discharge data precludes an a priori comparison of model performance.

## 2 METHODOLOGY

### 2.1 Data and catchments

This study uses a subset of the GAGES II reference basins. It can be therefore expected that they are only minimally affected by human disturbances. Daily discharge time series were downloaded from the USGS webpage, and all 574 catchments without discharge data gaps between October 1, 1981, and September 30, 2008 (27 years), were considered in this study.

The Daymet 2 dataset was used as source for the weather data (Thornton et al., 2014; Thornton, Running, & White, 1997). This dataset was chosen as a modelling study of U.S. catchments found that a previous Daymet version (Daymet 1) achieved a good performance (Newman et al., 2015). The 1980–2010 timeseries of the daily maximum and minimum temperatures (°C) and precipitation (mm d^−1^) were extracted for each catchment using the GAGES II catchment delineations. The potential evapotranspiration (PET), required as input to the hydrological models, was estimated according to Hargraeves and Samani (1982).

For learning about how catchments properties relate to model performance, the catchments were characterized with respect to climate, geology, landscape, topography, soil, and land use (Table 1). Summary statistics for the catchments are shown in Table 2. The catchments with a runoff coefficient larger than one were excluded from subsequent analyses after being identified as problematic in a screening step, which aimed at identifying the catchments that could not be properly modelled. Because there is no straightforward way of doing this, it was decided to focus on model performance with respect to bias. This identifies the catchments for which forcing data results in simulations that do not replicate well the long‐term water balance. Although the catchments passing this test are able to replicate the water balance, it must be noted that they still might have errors describing the short‐term dynamics. The implementation of this test resulted in the removal of 13 catchments. An analysis of the reasons for the poor performance of these catchments revealed that some of them had runoff coefficients larger than one, whereas the remaining had timing errors attributable to snow related problems.

**Table 1:** Catchment descriptors

Descriptors	Variables
Climate (C)	Mean annual and monthly precipitation, potential evapotranspiration, and temperature. Walsh seasonality ( Walsh & Lawler, 1981 ); Colwell's indices ( Colwell, 1974 ); maximum 1‐, 3‐, 7‐, 15‐, 30‐, 60‐, 90‐, and 120‐day precipitation and minimum 15‐, 30‐, 60‐, 90‐, and 120‐day precipitation; % of annual precipitation when temperature <0°C; number of days with precipitation when temperature <0°C; Koeppen climate zones (Kottek et al., 2006).
Geology/Lithology (G)	Hydraulic permeability ( Gleeson et al., 2011 ), 18 classes of surface lithology ( Sayre et al., 2009 ), baseflow index ( Wolock, 2003 )
Landscape/Morphology (M)	10 classes of land surface forms ( Sayre et al., 2009 ) and 5 classes of topographic moisture potential ( Sayre et al., 2009 )
Topography (T)	Elevation (mean, minimum, maximum, and range), slopes, and exposition predictors estimated from NED (National Elevation Dataset)
Soil (S)	138 soil properties (gSSURGO from Soil Survey Staff, 2015 )
Land use (L)	16 land use classes from the National Land Cover Database 2001 ( Homer et al., 2007 )

**Table 2:** Summary of catchment properties

Property	Unit	Min	Median	Max
Area	km ^2^	4	348	25,791
log k (permeability [m ^2^ ])	—	−16.48	−12.78	−10.87
Baseflow index (BFI a )	—	0.06	0.45	0.85
Available water capacity at 25‐cm depth	mm	0.18	3.65	6.68
Soil class A	% of basin	0	5	100
Soil class D	% of basin	0	10	98
Forest	% of basin	0	60	99
Crops/grass/pasture	% of basin	0	27	98
Wetland	% of basin	0	1	76
Mean elevation	m a.s.l.	9	472	3,644
Mean slope	°	0.0	6.5	33.6
Hypsometric integral	—	0.1	0.4	0.7
Plains	% of basin	0	29	100
Mountains	% of basin	0	6	88
Annual precipitation	mm	304	1,156	3,250
Annual PET b	mm	600	1,063	1,661
Walsh seasonality	—	0.05	0.22	0.89
Pp in winter index	—	0.3	0.9	2.1
Aridity index	—	0.24	0.96	4.65
Precipitation as snow	%	0	8	88
Mean annual discharge	mm	2	404	2,876
Runoff coefficient	—	0	0.36	0.98

a BFI from the USGS gridded dataset (Wolock, 2003).

b Evapotranspiration calculated with the Hargreaves formula.

### 2.2 Description of the model structures

The models used in this study were initially developed in the context of the work carried out by Atkinson et al. (2002), Farmer et al. (2003), and Jothityangkoon, Sivapalan, and Farmer (2001). They were then used with some minor modifications by Bai et al. (2009). For the present study, it was necessary to add a snow routine computing snow accumulation and melt in 100‐m elevation bands using a temperature‐index‐based approach.

**Figure 1:**
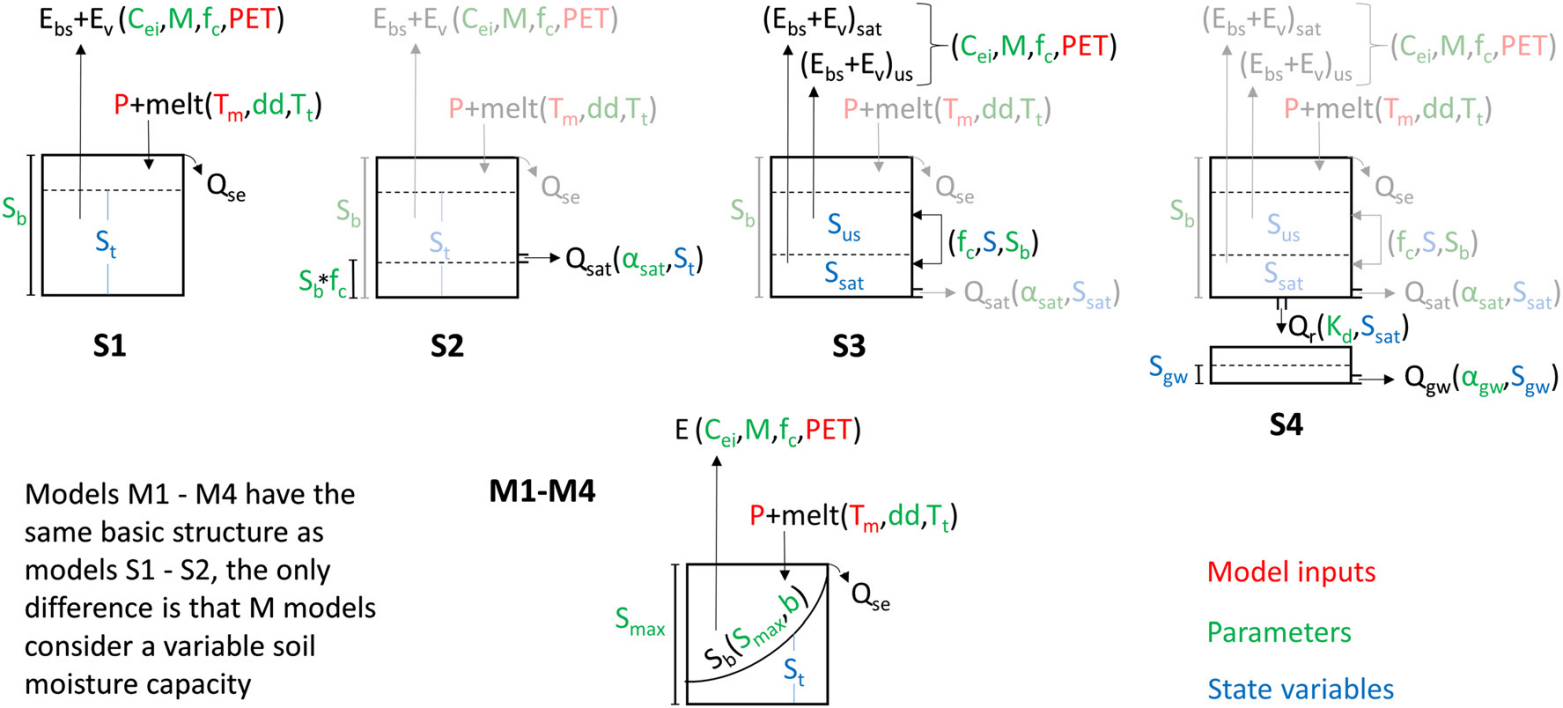
Sketch of model structures. All models have as input the precipitation (P), daily mean temperature (Tm), and potential evapotranspiration (PET) time series. Parameter names are in green (see table) and state variables in blue (St, level in soil storage; Sus, level in unsaturated compartment; Ssat, level in saturated compartment; Sgw, level in groundwater storage). The fluxes are in black: Ebs and Ev (evapotranspiration from bare soil and vegetated land); Qse, Qsat, and Qgw (discharge from excess overflow, saturated flow, and groundwater flow); and Qr (recharge)

The hydrological models are composed of different evapotranspiration, storage, and routing modules, which can be combined in various ways for testing alternative hypotheses about catchment behaviour (Figure 1). There are two evapotranspiration modules, both allowing for evaporation losses due to interception. Another feature shared by both modules is that they estimate separately the evapotranspiration for the fraction of the catchment covered by deep‐rooted vegetation and for bare soil areas. In this way, it is possible that the actual evapotranspiration equals the PET rate when soil moisture is above field capacity and the soil is covered by deep‐rooted vegetation. The difference between both modules is that the ET2 module distinguishes a saturated and an unsaturated zone in the soil bucket, whereas ET1 is composed by a single zone.

Model S1 consists of the evapotranspiration module ET1 and a soil storage producing discharge via saturation excess. Model S2 is additionally able to produce discharge through saturation flow once the water content in the soil storage is above field capacity. The evapotranspiration module ET2 is included in Models S3 and S4. The difference between these two models is an additional deep‐water bucket releasing groundwater in Model S4. Models M1 to M4 have the same structure as Models S1 to S4, but they differ in their assumptions about the distribution of the soil storage capacity in the landscape. The models from the S series assume a homogeneous storage and have thus a single bucket. On the other hand, the M models consist of 10 soil stores with varying depths, accounting in this way for variations in the soil storage capacity across catchments. The model equations and description of the parameters are provided in the Supporting Information.

An inspection of the model structures shows that there are two complexity axes: (a) the M/S axis, where the (multistore) M‐version of each model is considered more complex than its (single store) S‐version, and (b) the increase in complexity from Models 1 to 4 (i.e., S1 < S2 < S3 < S4 and M1 < M2 < M3 < M4). Although an overall summary of model performance for all models is presented in Section 3.1, the remaining analysis only considers one complexity axis and focuses therefore on the results obtained by Models S1 to S4 and Model M4.

The models were run for the period between the October 1, 1981, and September 30, 2008. As the first 3 years were for spinning up, the analysis was done for the remaining 24 years.

### 2.3 Monte Carlo modelling framework

The models were evaluated within a Monte Carlo (MC) framework. All parameters were sampled from a uniform distribution. The sampling ranges for the evapotranspiration, soil, and routing parameters were adopted from previous studies (Bai et al., 2009), whereas the ranges of snow accumulation threshold were taken from Rajagopal and Harpold (2016). The ranges of the snowmelt parameters were rather wide, accounting for the temperature biases that can be encountered in meteorological datasets (Behnke et al., 2016).

In an initial step, it was tested how many samples were needed for obtaining stable results. As 40,000 samples were found to provide robust estimates under repeat sampling, this number of parameter sets was drawn and then used for all models and catchments.

### 2.4 Hydrological signatures

Hydrological signatures characterize different aspects of the catchment response, in this case, the discharge, with respect to which models are assessed. The signatures used in this study are based on the set used by Jothityangkoon et al. (2001), Atkinson et al. (2002), and Farmer et al. (2003) for analysing the water balance at different timescales. For the sake of comparability with previous studies and as this set of metrics is still being used, although with some minor modifications (e.g., Bai et al., 2009; Coxon, Freer, Wagener, Odoni, & Clark, 2014), they were the first choice for the current analysis.

Sorted from longer to shorter timescales, the signatures are as follows:

- the interannual discharge variability, which is calculated as the total annual discharge (i.e., for a period of 24 years, there are 24 data points).
- Two signatures at the monthly timescale: the Pardé signature and the monthly time series. The first signature is based on the Pardé coefficients (Pardé, 1933), which are used for characterizing the flow regime (seasonal runoff????????). They are estimated as the ratio between the mean monthly discharge and the mean annual discharge (i.e., there are 12 data points, each representing the mean discharge for 1 month). The intra‐annual signature consists on the timeseries of the monthly discharge; this means that for a 24‐year period, there are 288 data points.
- Two signatures at the daily timescale: the discharge time series and the flow duration curve (FDC). Both signatures are based on the daily discharge, but it is expected that models are more successful at reproducing the FDC, as only the distribution of discharge—and not the timing—needs to be matched.

### 2.5 Evaluation measures

The Kling–Gupta efficiency (KGE) was used for quantifying model performance across these signatures. In other words, the KGE was used for assessing how well different MC runs are able to reproduce the signatures. It is calculated as (Gupta, Kling, Yilmaz, & Martinez, 2009):

(1) $$\mathrm{KGE}=1-\sqrt{{\left(r-1\right)}^{2}+{\left(\text{bias}\right)}^{2}+{\left(\text{stdev}\right)}^{2}}.$$

This metric is a combination of the bias, standard deviation, and correlation errors, each focusing on different aspects determining how well a model agrees with the observations. The bias and standard deviation error indicate how well models are able reproduce the mean and the variability of the discharge time series:

(2) $$\text{bias}=\left|\frac{{\bar{Q}}_{s}-{\bar{Q}}_{o}}{{\bar{Q}}_{o}}\right|\;,$$

(3) $$\text{stdev}=\left|\frac{\mathrm{std}\left(Q_{s}\right)-\mathrm{std}\left(Q_{o}\right)}{\mathrm{std}\left(Q_{o}\right)}\right|,$$

with ${\bar{Q}}_{s}$ and ${\bar{Q}}_{o}$ representing the simulated and observed mean discharge over the entire period and std(*Q*_*s*_) and std(*Q*_*o*_) standing for the standard deviation of the simulated and observed discharge time series, respectively. Pearson's correlation coefficient *r* characterizes the performance of the models regarding timing and shape. It can be seen that the KGE can reach a best value of 1 when the bias and standard deviation error are zero (i.e., the modelled and observed values are equal) and *r* is one.

### 2.6 Study set‐up

The first step aimed at replicating the conclusions of Atkinson et al. (2002), stating that more complex models are needed for more arid catchments and shorter timescales. Although Atkinson et al. (2002) relied on a visual assessment for determining which models had an “acceptable performance,” it was necessary to define a threshold for automatizing this task in the present study. The choice of a threshold will always be subjective (Diskin & Simon, 1977), but it should be goal‐oriented and take into account the available data (Guthke, 2017; Laiti et al., 2018). As this study aimed at comparing model performance at different timescales and for different model complexities, the threshold needs to take into account the abilities of the considered models at different timescales. Some test allowed to conclude that the best option was using three different thresholds. This prevents selecting a threshold that is too low, for example, at the interannual scale (meaning that almost all models would be accepted) but, at the same time, too high for models to be accepted at the daily timescale. There is a threshold for the daily timescale (KGE = 0.70), one for the FDC timescale (KGE = 0.85), and another for the annual and monthly timescales (KGE = 0.80). The ranking of the threshold values agrees with the KGE ranking of the timescales, as the best results were achieved for the FDC and the worst for the daily timescale. The threshold values are in between the lowest and highest median KGE values achieved with different models at each timescale (see Table 3), allowing to see the impact of using models with different complexities.

**Table 3:** Median KGE for the models at each timescale

	S1	S2	S3	S4	M1	M2	M3	M4
Interannual	0.73	0.77	0.75	0.78	0.74	0.78	0.76	0.78
Pardé	0.58	0.68	0.65	0.74	0.61	0.69	0.67	0.76
Intra‐annual	0.63	0.75	0.73	0.79	0.66	0.76	0.74	0.79
FDC	0.38	0.81	0.81	0.83	0.43	0.83	0.83	0.84
Daily	0.21	0.66	0.63	0.70	0.25	0.69	0.67	0.72

*Note.* The results show the average value for both validation periods.

The analysis was carried out for two periods comprising the first 12 and last 12 years, respectively. For each period, the MC run with the highest KGE was identified (calibration) and then the performance of this MC run was evaluated in the other period (validation). The mean results for both calibration periods are shown in the Supporting Information, whereas the mean results for the validation periods are presented in chapter 3.

The second step consisted in clustering the catchments for identifying the main factors influencing model performance. The rationale for this is that previous studies have reported that climate factors are the main determinants of model performance. As these factors (e.g., aridity, fraction of snow, and seasonality) vary independently from one another, it might be difficult to identify the impact of individual factors. By working with clusters, it is possible to carry out a two‐stage analysis first establishing which “first‐order factors” separate the catchments into different performance clusters. In the second stage, it is then analysed how the variations in performance relate to changes in climate and catchment properties in each cluster.

It must be considered that it is possible to cluster the catchments according to many factors, for instance, climate and catchment properties, the value of signatures, or model performance. The results of such an exercise will also vary depending on the selected clustering algorithm, its parameterization, and the considered catchments. This indicates that this clustering of catchments into different groups will never be “objective” and that we should aim at a classification that is reproducible and adequate for fulfilling its intended purpose. In this study, the clusters were defined manually on the basis of maps of model performance and the distribution of predictors. Although manual clustering is not among the most commonly clustering approaches used in hydrology, there are examples of hydrological studies relying on this technique (e.g., Berghuijs, Sivapalan, Woods, & Savenije, 2014).

After a visual analysis of the performance maps, it was decided to cluster the catchments into four groups. The borders of the clusters were defined on the basis of catchment properties. A map was drawn with the distribution of each catchment property, and it was then visually assessed if the spatial distribution of the properties correlates with the distribution of one or more performance clusters. If this was the case, the threshold for delineating the clusters was searched by testing different thresholds for the catchment properties and seeing how well they agreed with the model performance patterns. The catchment property and corresponding threshold that separated the catchments into groups with homogenous performance were then used for defining the borders of the clusters. It is important to stress that these thresholds were only identified for facilitating the reproducibility of the clustering and that they are not used in the analysis of the results.

## 3 RESULTS

### 3.1 Median performance of the models across the catchments

Table 3 shows the performance achieved by the models at different timescales. It is seen that there are only small performance differences between the performance of the M‐ and S‐model structures when considering Models 2, 3, and 4, whereas there are larger differences between the S1 and M1 models, especially for the FDC and daily signatures.

With respect to the performance across timescales, the daily signature is the most difficult to simulate, whereas the FDC is the signature that can be modelled best, at least for Models 2, 3, and 4. The situation is different for Model 1, which only produces discharge through the excess overflow mechanism (i.e., there is only runoff when the soil storage is full, but there is a continuous loss through evapotranspiration), which means that there are large errors at the daily timescale affecting the ability of this model for reproducing the FDC. This is why Model 1 is more successful at modelling the annual, monthly, and Pardé signatures instead.

An analysis of the variability of model performance across timescales shows that the differences between models tend to be more pronounced at the daily scale than at the monthly or annual scales. For example, the KGE difference between the model with best and worst performance is about 0.5 at the daily timescale, 0.16 at the intra‐annual timescale, and about 0.05 at the annual scale. This variation in performance depends on the complexity of the considered model, as more complex models have more homogenous results across timescales than simpler models. This can be seen for instance, when looking at the KGE difference between the daily and annual timescales, which equals 0.52 for Model S1, about 0.12 for Models S2 and S3, and 0.08 for Model M4.

### 3.2 Assessment of the adequacy of using the aridity index a performance predictor

**Figure 2:**
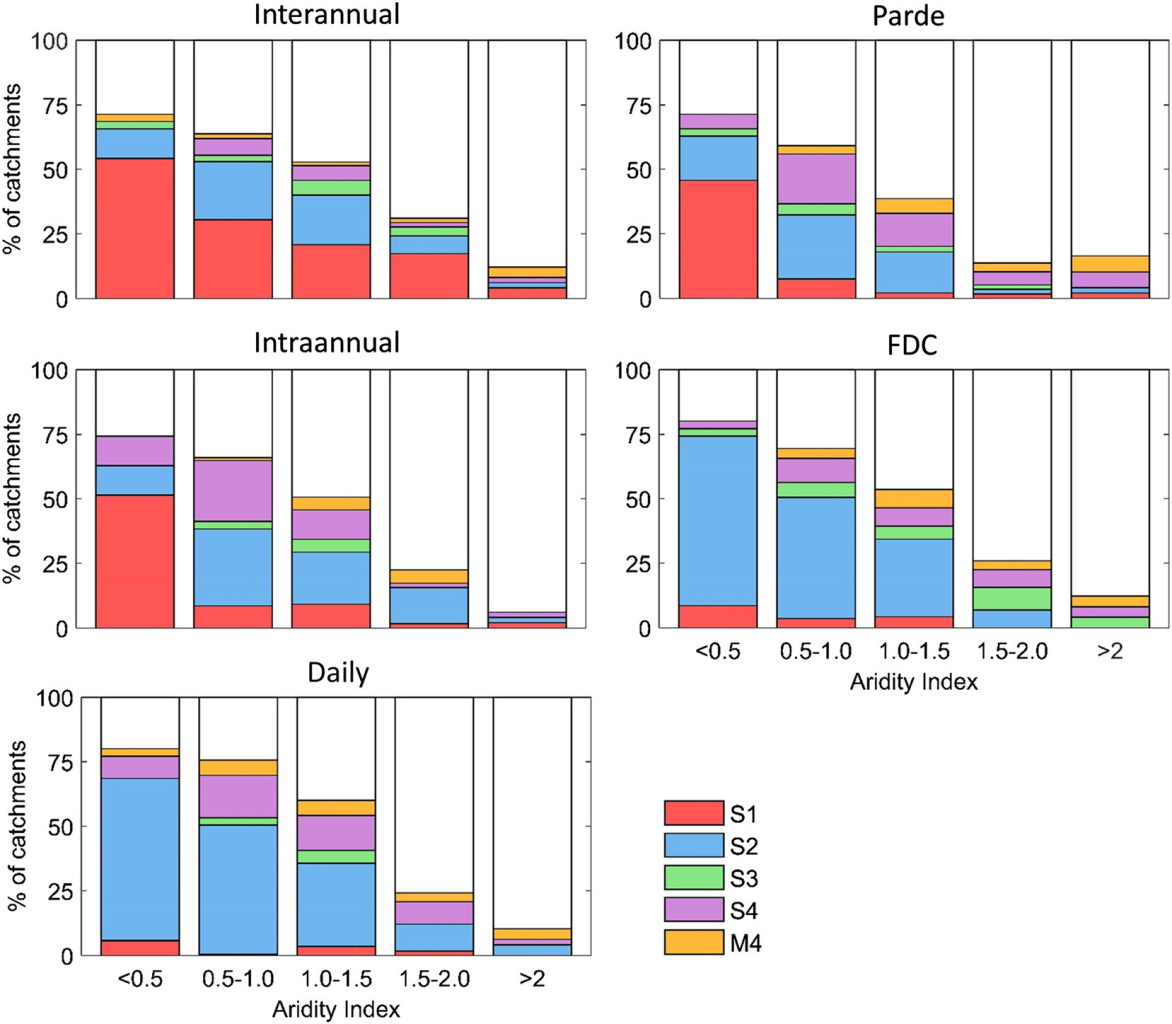
Percentage of catchments for which each model is the simplest one reaching the performance threshold at each timescale disaggregated by aridity classes. The acceptance thresholds are 0.80 KGE for the interannual, intra‐annual, and Pardé signatures, 0.85 for the FDC, and 0.70 for the daily timescale. The white space shows the percentage of catchments for which no model achieves the chosen threshold

This section investigates if the aridity index can be related to model performance as shown in Atkinson et al. (2002) who found that arid catchments require more complex models for achieving acceptable performances. Figure 2 shows the fraction of catchments for which each model is the simplest one achieving a performance above the threshold. The target, for example, of the Pardé signature in the second aridity class (i.e., aridity index 0.5–1.0), is reached with Model S1 by about 7% of the catchments, with Model S2 by 25%, with Model S3 by 4%, with Model S4 by 20%, and with Model M4 by 3%. The remaining 41% of catchments do not achieve a performance above the threshold with any model.

In general, it is possible to identify the patterns mentioned by Atkinson et al. (2002). It can be seen, for example, that the Pardé and interannual signatures can be modelled with the simplest model in around 50% of the wettest catchments, but that this simple model is not able to achieve acceptable results in more arid catchments. For the FDC, it is further seen that the relative importance of the two most complex models increases continuously as the catchments get dryer. For the wettest catchments, for instance, Models S4 and M4 are responsible for about 3% of the catchments reaching the performance threshold. This percentage increases to 19%, 26%, 40%, and 66% with increasing aridity. A consistent feature across all signatures is the decrease in the fraction of catchments achieving the performance targets as catchments become dryer.

On the other hand, there are also cases in which the results are not aligned with the findings of Atkinson et al. (2002). An example of this is the lack of an impact of Model M4 when reproducing the intra‐annual signature in the driest catchments.

### 3.3 Identification of spatial patterns in model performance

The results presented in the previous section highlight that aridity is an important factor determining model performance across catchments. This section explores the impact of other catchment and climate properties, besides aridity, on model performance. This is done by separating the United States into zones with similar model performance and analysing each of these clusters separately.

**Figure 3:**
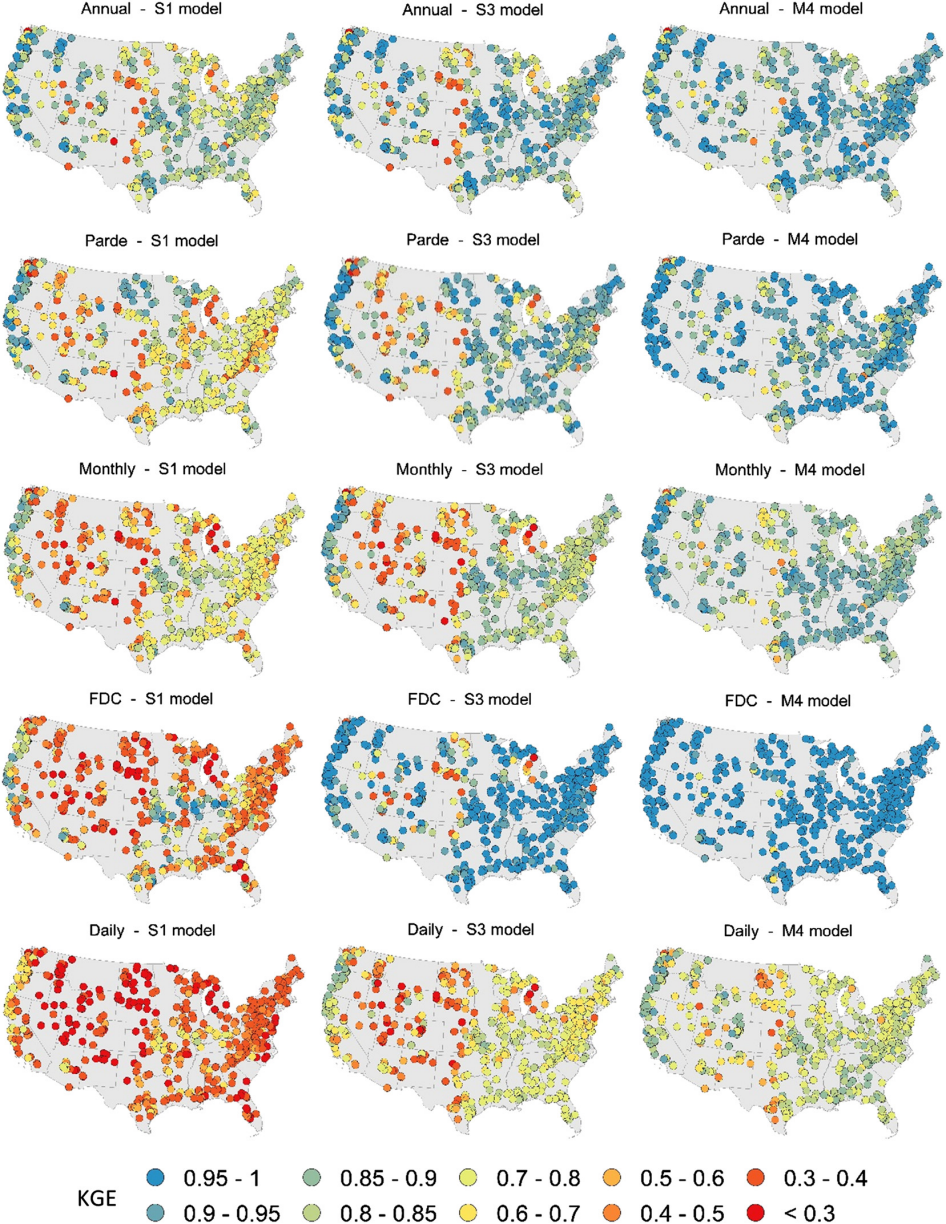
Maps showing the best KGE obtained for each timescale with Models S1, S3, and M4

**Figure 4:**
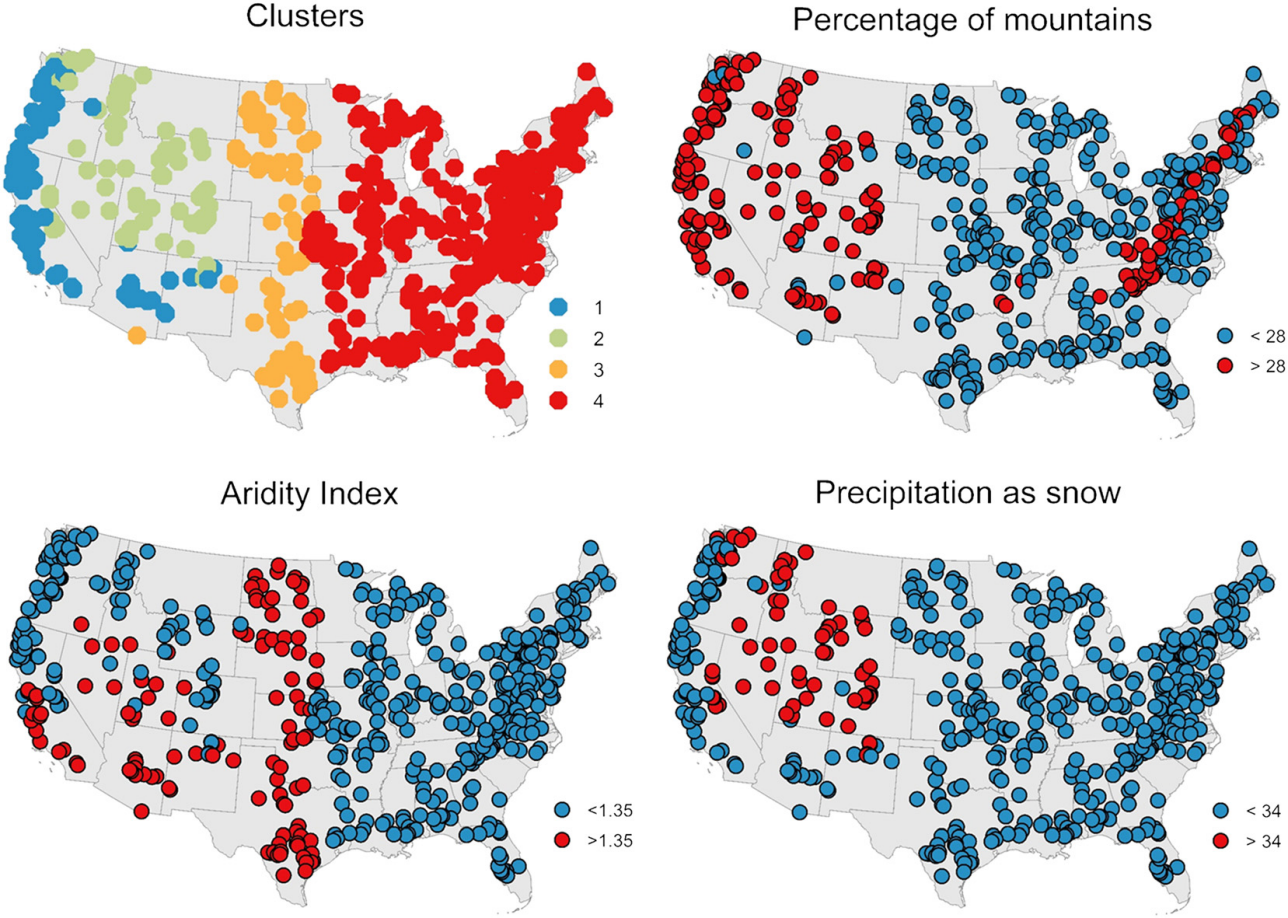
Location of the catchments in the four clusters and location of catchments above the threshold for the percentage of mountains in the catchments (28%), the fraction of precipitation falling as snow (34%), and the aridity index (1.35)

Figure 3 presents the best performance obtained with Models S1, S3, and M4 for each timescale in a series of maps. A pattern that can be identified at each timescale, especially using Model S3, is the variation in model performance from West to East: Model performance decreases from the West coast to the interior until the central part, from which on performance tends to increase again towards the East. This pattern was used for clustering the catchments into four groups. In the first step, two clusters were built by separating the western from the eastern catchments. In the second step, each of these groups was separated again into two, totalling four clusters (Figure 4). The clusters were delineated on the basis of the value of three catchment predictors selected according to the procedure described in the methodology, that is, by comparing the spatial patterns of maps with catchment properties with the spatial patterns of model performance maps. The separation of the catchments into a western and an eastern cluster was carried out on the basis of the percentage of mountains (according to the definition of Sayre, Comer, Harumi, & Jill, 2009) in the catchments. A threshold of 28% separated the catchments into two groups that agreed well with the performance patterns in Figure 3: the western catchments with a higher percentage of mountains and the flatter catchments in the east. The catchments located in the Appalachian Mountains, exhibiting a high coverage of mountains, were not considered as a separate cluster, as they do not seem to have a different performance than the surrounding catchments (Figure 3). In a second step, a threshold of 34% of precipitation falling as snow was selected for separating the catchments with a high fraction of mountains into Clusters 1 (low importance of snow) and 2 (high importance of snow). Finally, Clusters 3 and 4 were created by splitting the cluster with a low fraction of mountains based on an aridity index threshold of 1.35.

With respect to the predictors used for defining the clusters, there are many studies highlighting the impact of aridity and snow on model performance (e.g., Berghuijs et al., 2014). For the importance of mountains, the situation is different, as it is not regarded a main factor affecting directly neither the hydrologic signatures nor model performance. Landscape is, however, recognized as an important factor in soil formation (Blume et al., 2010); it is expected to affect surface and subsurface water flow (Sawicz, Wagener, Sivapalan, Troch, & Carrillo, 2011; Winter, 2001) and has an influence on climate (Siler, Roe, & Durran, 2013). It might therefore be argued that mountains are closely related to the snow in Cluster 2 and that the higher precipitation in the Western coast (Cluster 1) might be enhanced by orographic precipitation (Roe et al., 2003) justifying in this way the use of this predictor for defining the clusters.

### 3.4 Model performance for the clusters

**Figure 5:**
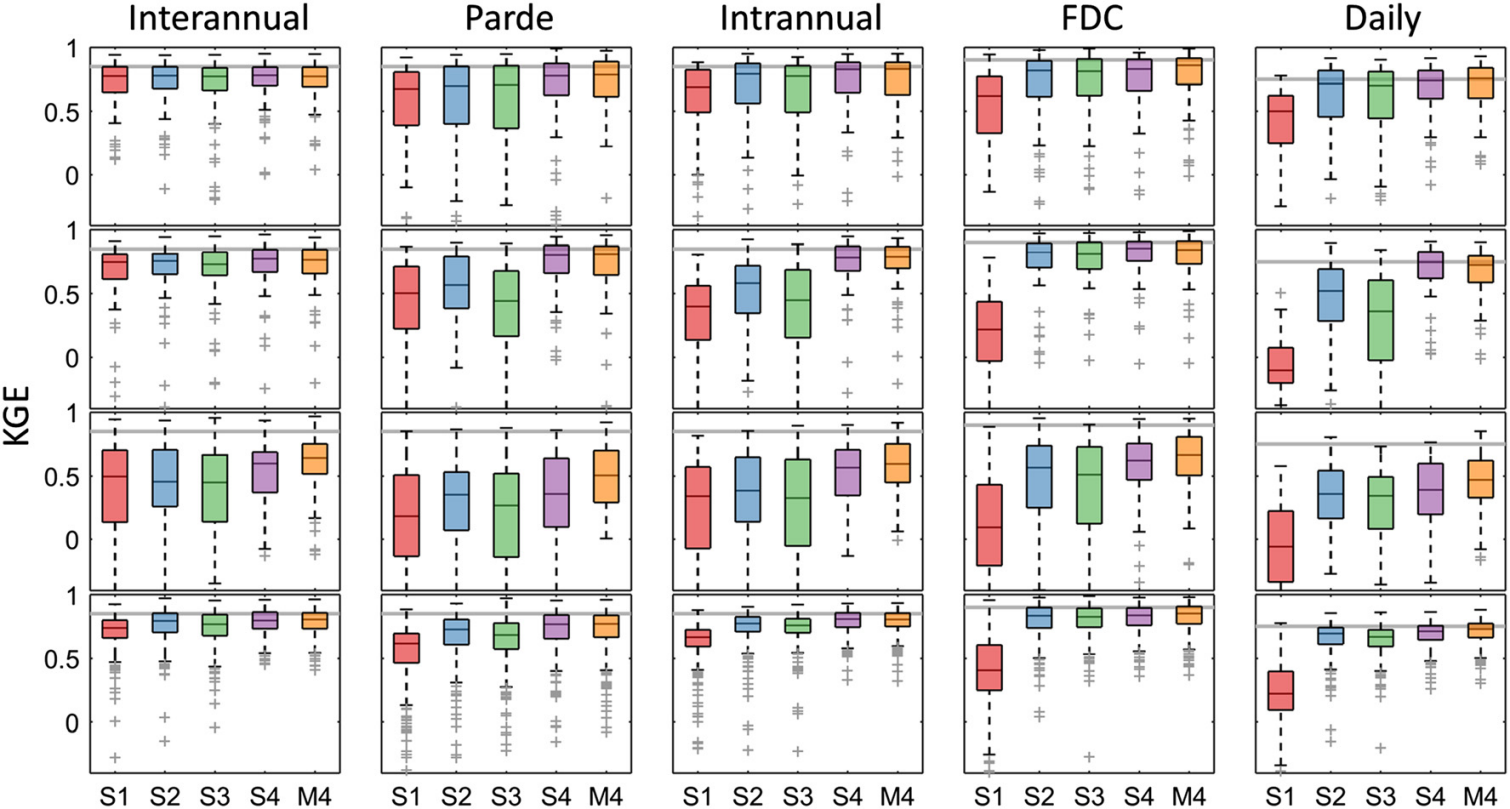
Boxplots with the KGE performance of Models S1, S2, S3, S4, and M4 for each cluster and timescale. The light grey line identifies the performance threshold of 0.85 KGE. There is one outlier catchment now shown here for Model S1 at the FDC and daily timescales and two outliers not shown for the Pardé and interannual signatures for Models S1, S2, and S3

Figure 5 shows a summary of model performance in each cluster and for each timescale. There are clear differences between clusters, especially for shorter timescales and simpler models. In agreement with the maps in Figure 3, Clusters 1 and 4 tend to have better performances than snowy (Cluster 2) and arid (Cluster 3) catchments at all timescales.

**Figure 6:**
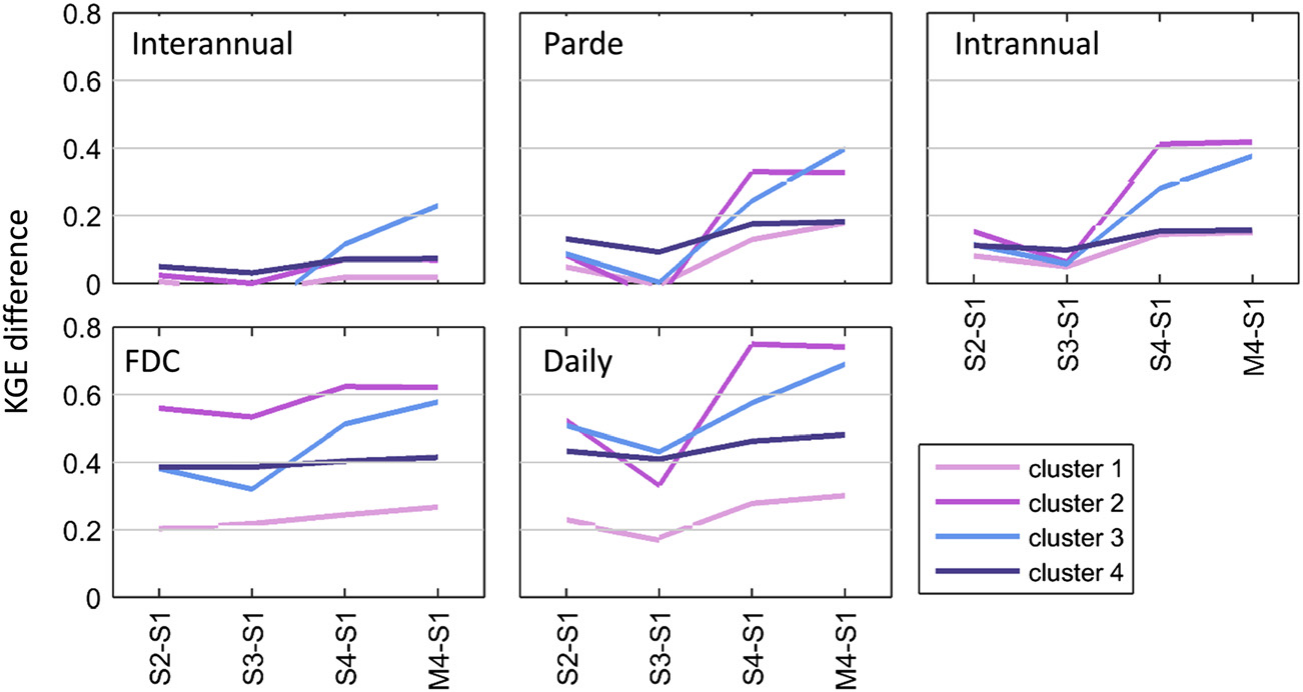
Average difference in performance (KGE) achieved if Models S2, S3, S4, and M4 are used instead of Model S1. Analysis for all clusters and timescales

Figure 6 shows the changes in average performance when using Models S2, S3, S4, and M4 instead of Model S1. For all clusters, the impact of using more complex models increases with smaller timescales. For example, the largest performance increase at the interannual scale is about 0.18 KGE (with Model M4), rising to about 0.8 at the daily timescale.

We further see that more complex models have a much smaller potential for improving the performance in Clusters 1 and 4, while being especially successful at improving the performance in snowy catchments (Cluster 2). A comparison of the increase in performance achieved when going from Model S4 to M4 (compare S4–S1 with M4–S1) shows that Cluster 3 (arid) benefits most from a shift to the most complex model.

Overall, Models S3 and M4 show much smaller increases in performance than Models S2 and S4. In some cases, Models S3 and M4 even have a lower performance than Models S2 and S4, respectively. Model S3 should be avoided for modelling snowy catchments, as its performance is considerably lower than the one for Model S2. These catchments should instead be modelled with Model S4, which clearly has the best performance for this cluster.

### 3.5 Identifying local predictors important for each cluster

**Figure 7:**
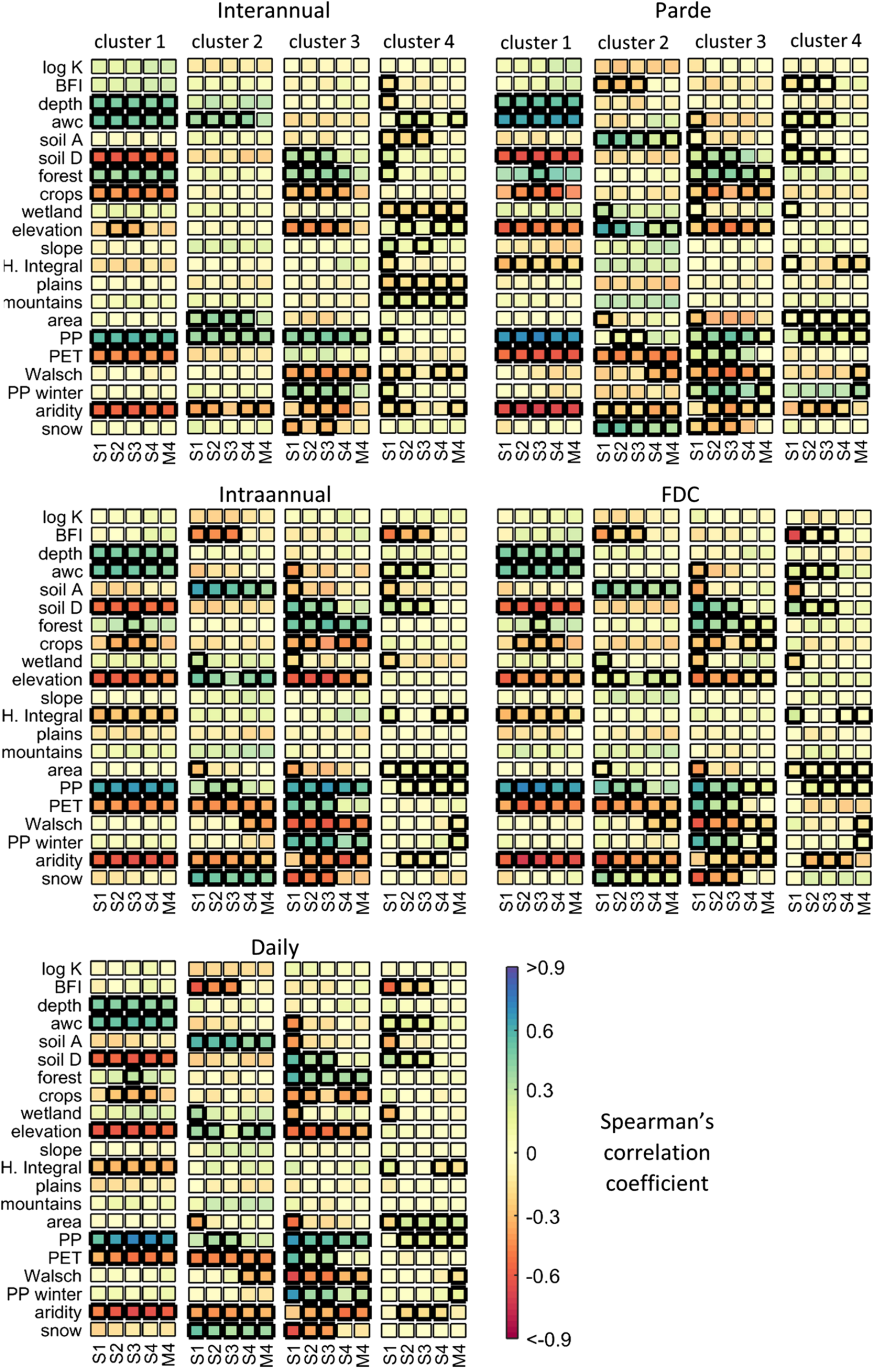
Spearman's correlation between 21 predictors and model performance (KGE) for each timescale, cluster, and Models S1 to S4 and M4. Values with a thick black border are significant (*p* < .01)

The next step is to identify relationships between the predictors and model performance in each cluster. This was done using Spearman's correlation coefficient between the value of the predictors and the KGE. The thick black borders in Figure 7 highlight the relationships with a significant correlation (*p* < .01). It is interesting to note that, for some predictors, the sign of the correlation varies with the cluster, for example, the relationship of elevation and snow in Clusters 1 and 2. This explains the difficulties in finding overall relationships between performance and predictors when considering all catchments together. Another finding is that there correlations are in general highly consistent across timescales.

**Figure 8:**
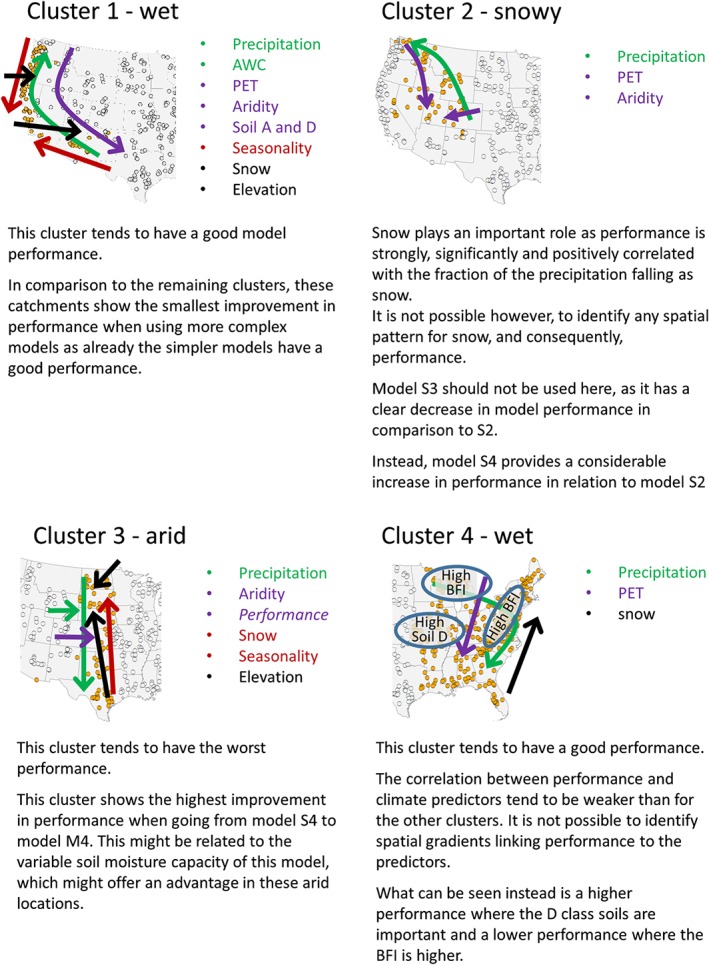
Summary plot for the clusters. The arrows always point in the direction in which there is an increase in the values of the considered variables

The next paragraphs provide a brief summary of the way in which the predictors vary in each cluster and relates this to model performance. This description is supported by some maps showing the most important relationships (Supporting Information) and a graphic summary (Figure 8).

In Cluster 1, model performance and precipitation have a positive and significant correlation for all signatures. Precipitation, and therefore model performance, is lowest for the interior catchments in the Southeast and increases towards the Northwest (see Figure 8). The PET, on the other hand, increases from North to South exhibiting no longitudinal variations. Because aridity is estimated as the quotient of the PET and the precipitation, this results in a clear increasing trend of the aridity from North to South. Other predictor with a high, although negative, correlation is the elevation, which increases from the coast to the interior and also reaches its highest values in the catchments located in the Southeast. Some soil properties also correlate with performance. A look at their spatial distribution shows that soils in the north tend have a higher available water capacity and a lower fraction of the catchment with soil in the D (clayey) and A (sandy) classes, suggesting that they have a higher proportion of loamy and silty soils, which are the ones with largest water holding capacity. Because climate is a main factor affecting soil formation, it is not unreasonable to assume that soils in the northern part are the result of the wetter conditions (coevolution). It is thus not possible to know if performance is primarily affected by the soil class or the climate; it is only possible to say that there is a significant correlation of these properties with model performance. Finally, there is a negative correlation between performance and the baseflow index (BFI), which is, however, not easily spotted by looking at the maps.

In Cluster 2, performance is positively correlated with elevation and snow. Because these predictors show no spatial pattern, they are not shown in Figure 8. There is also a positive correlation with precipitation, which decreases from NW to SE, and a negative correlation with the PET, which is more pronounced in the North and Eastern catchments and agrees well with the patterns for the aridity index. Model performance also tends to be negatively correlated with the BFI and positively correlated with the percentage soil in Class A. It was, however, not possible to identify spatial patterns for these properties.

For Cluster 3, elevation tends to increase from South to North and from East to West. Because elevation correlates negatively with model performance for this cluster, we see an increase of performance towards the South and the East. Precipitation and the PET, on the other hand, increase towards the Mexican Gulf, showing a positive correlation with performance. It was not possible to see an East–West pattern for the PET, but the increase in precipitation towards the east nevertheless results in an eastwards decrease of aridity.

For Cluster 4, there is an increase in PET and also an increase in precipitation to the south, which results in a small variability of aridity in this cluster. Snow plays an important role in only few catchments in the north, which might explain its erratic correlation for different timescales. There is further a correlation of model performance with the fraction of Class D soil and the BFI (see Figure 8).

## 4 DISCUSSION

### 4.1 Relationship between model performance, model complexity, and timescale

The question of choosing a model of adequate performance has attracted much interest in hydrology. Although it is expected that more complex models can achieve better performances than simpler models, there are different reasons why this might not be the case. Perrin, Michel, and Andreassian (2001) concluded from an analysis in 429 catchments with 20 model structures that more complex models might provide better results during calibration—due to overfitting—but that models with more than three to five parameters did not have a better performance than simpler models during validation. This agrees well with the findings of Jakeman and Hornberger (1993) who estimated that only about three parameters could be identified in a conceptual rainfall–runoff model when only discharge data are available for calibration. Performance, and the supported model complexity, will therefore be different whether we consider the models in calibration or validation mode.

Another reason why more complex models might not give better results than simpler ones is that the increased complexity might not address the reasons for the poor performance of a simpler model. This is a limitation also in this study, as it relies on eight already developed models that were applied in all catchments. Because of the large number of catchments, it was not possible to carry out the analysis as done by Atkinson et al. (2002), who could evaluate each model individually, identify the processes that might be limiting the results in each case, and then propose a model with increased complexity, which addressed these deficiencies. Instead, this study looked at models with increasing complexity, but it is well possible that the additional process (es) considered in more complex models are not responsible for the reduced performance of the simpler model. This seems to happen with Model S3, which in general does not improve performance but even decreases it in some cases (Figure 6). Future studies using these models could therefore compare the performance of Model S4 with a new model adding a groundwater storage to the S2 structure, which might give better results than Model S4, which combines Model S3 with a groundwater storage.

With respect to the timescale, this study confirms that it is in general easier to obtain good modelling results at more aggregated timescales (Atkinson et al., 2002; Bai et al., 2009; Chiew, Stewardson, & McMahon, 1993). The reason for this is that signatures at higher time resolutions need to account for the short‐term flow dynamics, something that often requires the use of more complex models. For lower temporal resolutions, we look at aggregated results and could, therefore, have a low ability for modelling the short‐term fluctuations but still get the average right. This has implications for model comparison suggesting that for understanding and being able to appreciate the differences between models, it is necessary to analyse them at daily (or smaller) timescales. It further explains why the KGE values obtained for the different models are more homogeneous at the interannual timescale than at the daily scale (Table 3 and Figure 5).

### 4.2 Relationship between model performance and catchment properties

There are numerous studies unveiling the relationships between model performance and catchment predictors. The most commonly identified relationship is with catchment aridity, which is found to correlate negatively with model performance (Gudmundsson, Wagener, Tallaksen, & Engeland, 2012; McMahon et al., 2013; Parajka et al., 2013; Parajka et al., 2013; van Esse et al., 2013; Weingartner et al., 2013). This study agrees with this, as there is a consistent negative correlation between performance and aridity for all clusters. Viglione et al. (2013) hypothesize that the reduction of performance in arid catchments might be caused by an increasing nonlinearity of the runoff processes in arid catchments, as in dryer catchments rainfall input and runoff might only be coupled for wetter conditions or high rainfall intensities. In addition, rainfall events are more episodic in arid catchments, making them less predictable and more difficult to model (Pilgrim, Chapman, & Doran, 1988). Another explanation might be that an adequate treatment of soil processes becomes more critical for achieving a good performance in more arid catchments. This is because the main task of a model in a wet catchment is to route the water to the outlet, which can be done in many different ways, resulting in a higher degree of flexibility. If there is no, or only a little input of water into the system, it is critical to model what happens to this water for being able to reproduce the hydrograph correctly. This task is hampered by our limited knowledge about the subsurface properties, especially the water holding capacity, which is critical for an adequate representation of these processes (Atkinson et al., 2002).

The importance of snow has also been identified as a factor affecting model performance. More specifically, model performance tends to improve with increasing impact of snow (Merz, Parajka, & Blöschl, 2009). One possible explanation for this relates to the use of day degree melt approaches in lumped models. Because snowmelt depends in this case on the temperature, which is a variable with lower uncertainty than precipitation, we also expect a more reliable output. Another reason is that snowmelt produces a distinct peak which results in a seasonally varying hydrograph. If models are evaluated with the Nash–Sutcliffe efficiency, it is easier to achieve a good performance when there is a seasonal signal in comparison with a catchment with more uniform discharge across the year (Schaefli & Gupta, 2007). In this study, we found that the sign of the correlation for the fraction of snow varies depending on the cluster. Although snowy catchments (Cluster 2) have a positive correlation with performance, this is not the case for the other three clusters. However, as snow does not play an important role in most catchments assigned to the nonsnowy cluster, it is not meaningful to estimate this correlation for those clusters.

The relationship between seasonality and model performance is difficult to assess due to the multiple ways that exist for defining and estimating seasonality (e.g., Berghuijs & Woods, 2016; Laaha & Blöschl, 2006). However, it is expected that, as in the case of snowy catchments, the seasonal variability of the hydrograph affects the ease with which high NSE values can be achieved.

With respect to the subsurface properties, this study found similar results to Coxon et al. (2014), who found that catchments with a lower BFI (less groundwater dominated) tend to be easier to model than catchments in which groundwater plays a more important role. Although this might be partially explained by the lack of consideration of chalk‐related processes the models, which were important in the catchments with high BFI in the Coxon et al. (2014) study, it is also possible that the limited knowledge and consideration of subsurface processes have a larger impact on the catchments with higher importance of subsurface flow.

Studies linking catchment area and slope to model performance do not allow reaching a clear conclusion about these issues. McMahon et al. (2013), Weingartner et al. (2013), and Castellarin et al. (2013) were not able to establish clear relationships between model performance and catchment area. Although Parajka, Viglione, et al. (2013), Merz et al. (2009), van Esse et al. (2013), and Poncelet et al. (2017) found that performance improves with catchment area. This relation is, however, not that clear, as Merz et al. (2009) found no clear trend for catchments smaller than 2,000 km^2^ and only a weak relationship for larger catchments. This positive correlation was attributed to the increasing number of meteorological stations considered in larger catchments, which is reflected in a reduced uncertainty in the forcing data. In this study, we found in some cases a negative and in other a positive correlation with area. An analysis of the spatial distribution of the catchment sizes showed that the large catchments in this dataset tend to be concentrated in the central, arid part of the United States, which exhibits a lower performance. As we expect that aridity will have a larger influence on model performance than catchment area, it does not seem helpful to estimate the correlation with area for the whole group of catchments together, as the results will reflect the impact of aridity rather than catchment size. With respect to slope, Parajka, Andréassian, et al. (2013) hypothesized that its relationship with model performance depends on how aridity varies with elevation (and therefore slope, which correlates with elevation).

This study also identified some relationships between soil properties and model performance. Soil depth has a consistent positive correlation with model performance across clusters, models, and timescales. The percentage of soil in Classes A and D also has a significant relationship with performance in many clusters and timescales. These relationships are, however, not consistent between the clusters as the correlation is, in some cases, positive and, in others, negative.

### 4.3 Model selection based on catchment properties

Being able to identify an adequate model based on the catchment properties is an important goal in hydrology. Although not an easy task (Ley, Hellebrand, Casper, & Fenicia, 2016; van Esse et al., 2013), there are some starting points that can guide further studies in this area.

The first important thing we have learnt is that, although being counterintuitive, climate properties might be more critical for selecting adequate model parameters (Merz, Parajka, & Blöschl, 2011; Rosero et al., 2010) and model structures (van Esse et al., 2013) than nonclimatic factors. Considering that model structure describes the physical properties of the catchments, we would have expected that soil, geological, and landscape features (rather than climatic properties) would determine the model structure to use. A catchment‐related predictor that could however affect the ease for finding an adequate model structure and parameterization is the BFI, as various studies have found that groundwater dominated catchments require more tailored model structures (Coxon et al., 2014; van Esse et al., 2013).

The second point is that the catchment properties related to model performance, as well as the direction of this relationship (i.e., positive or negative correlation), might show large variations depending on the considered catchments. In this study, we found some properties exhibiting a consistent relationship with model performance for all considered zones, for example, aridity, whereas the impact of other properties varies with the zone. The properties showing a consistent behaviour can be then considered as first‐order predictors of model performance. Other properties might (or might not) be important, depending on the considered location. It must also be considered that the observed hydrograph is the result of the interplay between many different properties (and the processes each property affects), making it difficult to isolate the impact of individual properties.

## 5 CONCLUSIONS

Focusing on the performance of models with increasing complexity and linking this to catchment predictors is a good way for identifying which factors contribute to making a catchment “easier” or “more difficult” to model. Catchments that are easier to model achieve acceptable results with a large range of models and give us more flexibility when deciding which model to use, also making this decision less relevant. On the other hand, when dealing with catchments that are harder to model, we face more constrains in the number of models we can use and will probably need to invest more effort in modelling for obtaining a satisfactory performance (Clark et al., 2008).

One important finding of this study is that the relationship between catchment predictors and model performance suggested by Atkinson et al. (2002) is also valid for the catchments used in this study. The results further agree with previous studies regarding (a) the predictors identified as important and the (b) relationship between performance and timescale. With respect to the relevant predictors, it was seen that the BFI and the aridity index result in consistent performance patterns across all clusters and timescales. They are both negatively correlated with performance, that is, arid and groundwater‐dominated catchments tend to have lower performances. It was also seen that snow is a relevant predictor in areas with a large impact of snow. Another predictor with consistent performance is the soil depth, which correlates positively with performance. Many of these predictors are related to the subsurface properties, indicating that our lack of knowledge about the subsurface characteristics as well as the difficulties involved in “seeing” what happens below the surface are critical factors for improving our models. Regarding the timescales, it seems easier to get acceptable model results for longer than for shorter timescales. It was also seen that the benefit of more complex models becomes evident at shorter timescales.

A limitation of this study is that the results are not easily extrapolated to other hydrological models. Future studies could therefore carry out similar analyses for modular models (e.g., Clark et al., 2008; Fenicia, Kavetski, & Savenije, 2011), which might allow to link the differences in performance to specific structural units. Another route that could be taken by future studies is to determine the complexity of the catchments (e.g., Pande & Moayeri, 2018) and then link this metric to model performance.

## DATA AVAILABLITY STATEMENT

This study uses publicly available datasets as described in the methodology. Intermediate results supporting the findings of this study are available from the corresponding author upon reasonable request.

## Supporting information

Data S1. Median KGE for the models at each timescale when considering the MC run with highest KGE value for each catchment

Data S2 Supporting Information

Data S3 Supporting Information

Data S4 Supporting Information

Data S5 Supporting Information

Data S6 Supporting Information

Data S7 Supporting Information

Data S8 Supporting Information

Data S9 Supporting Information

Data S10 Supporting Information

Data S11 Supporting Information

Data S12 Supporting Information

Data S13 Supporting Information

Data S14 Supporting Information

Data S15 Supporting Information

Data S16 Supporting Information
