# Supplementary material for: Identification of factors influencing hydrologic model performance using a top‐down approach in a large number of U.S. catchments
Source: Hydrol Process. 2019 Nov 5;34(1):4–20. doi: 10.1002/hyp.13566 (PMC6973287; doi:10.1002/hyp.13566)
Supplement: Supplementary file 1 — Data S1. Median KGE for the models at each timescale when considering the MC run with highest KGE value for each catchment [file HYP-34-4-s001.docx]

*Table A1: Median KGE for the models at each timescale when considering the MC run with highest KGE value for each catchment*

|  | **S1** | **S2** | **S3** | **S4** | **M1** | **M2** | **M3** | **M4** |
| --- | --- | --- | --- | --- | --- | --- | --- | --- |
| **Interannual** | 0.85 | 0.91 | 0.91 | 0.94 | 0.86 | 0.91 | 0.92 | 0.94 |
| **Pardé** | 0.74 | 0.88 | 0.88 | 0.94 | 0.77 | 0.88 | 0.88 | 0.94 |
| **Intraannual** | 0.71 | 0.84 | 0.83 | 0.89 | 0.75 | 0.84 | 0.84 | 0.90 |
| **FDC** | 0.47 | 0.96 | 0.97 | 0.98 | 0.52 | 0.98 | 0.98 | 0.80 |
| **Daily** | 0.28 | 0.75 | 0.73 | 0.79 | 0.34 | 0.77 | 0.75 | 0.79 |
