# Supplementary figures and images for: Identification of factors influencing hydrologic model performance using a top‐down approach in a large number of U.S. catchments
Source: Hydrol Process. 2019 Nov 5;34(1):4–20. doi: 10.1002/hyp.13566 (PMC6973287; doi:10.1002/hyp.13566)

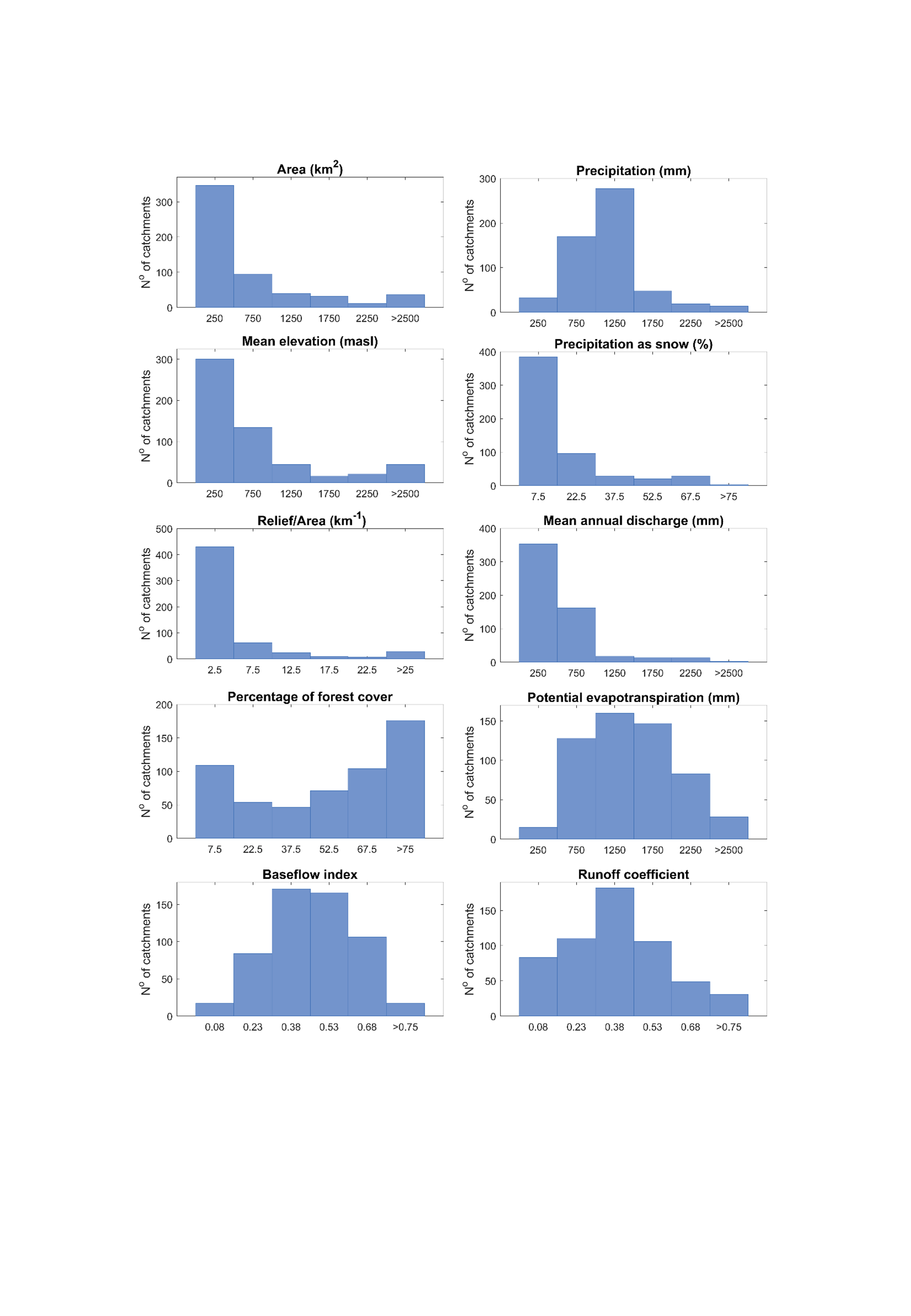

Supplement: Supplementary file 2 — Data S2 Supporting Information [file HYP-34-4-s002.tif]

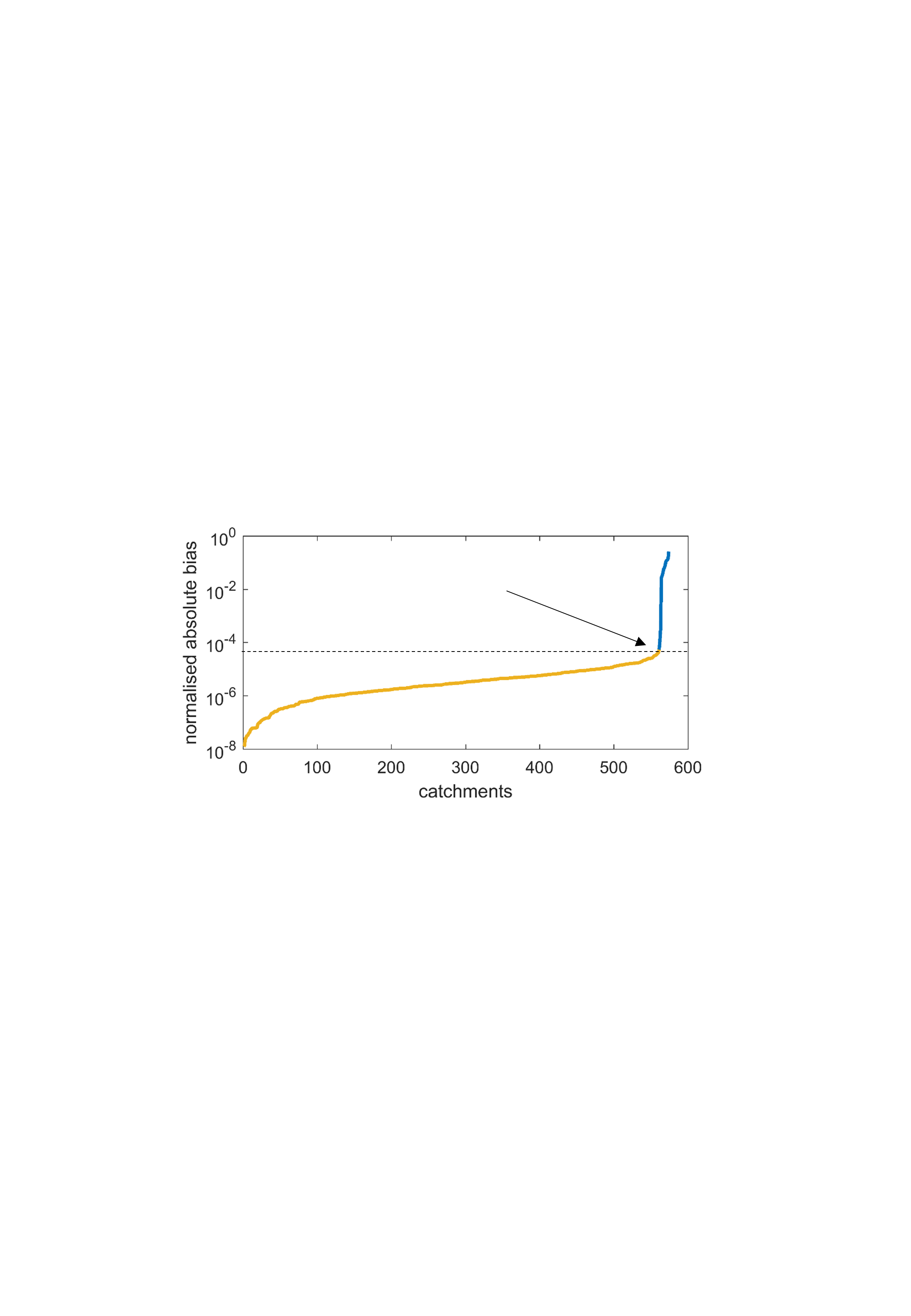

Supplement: Supplementary file 3 — Data S3 Supporting Information [file HYP-34-4-s003.tif]

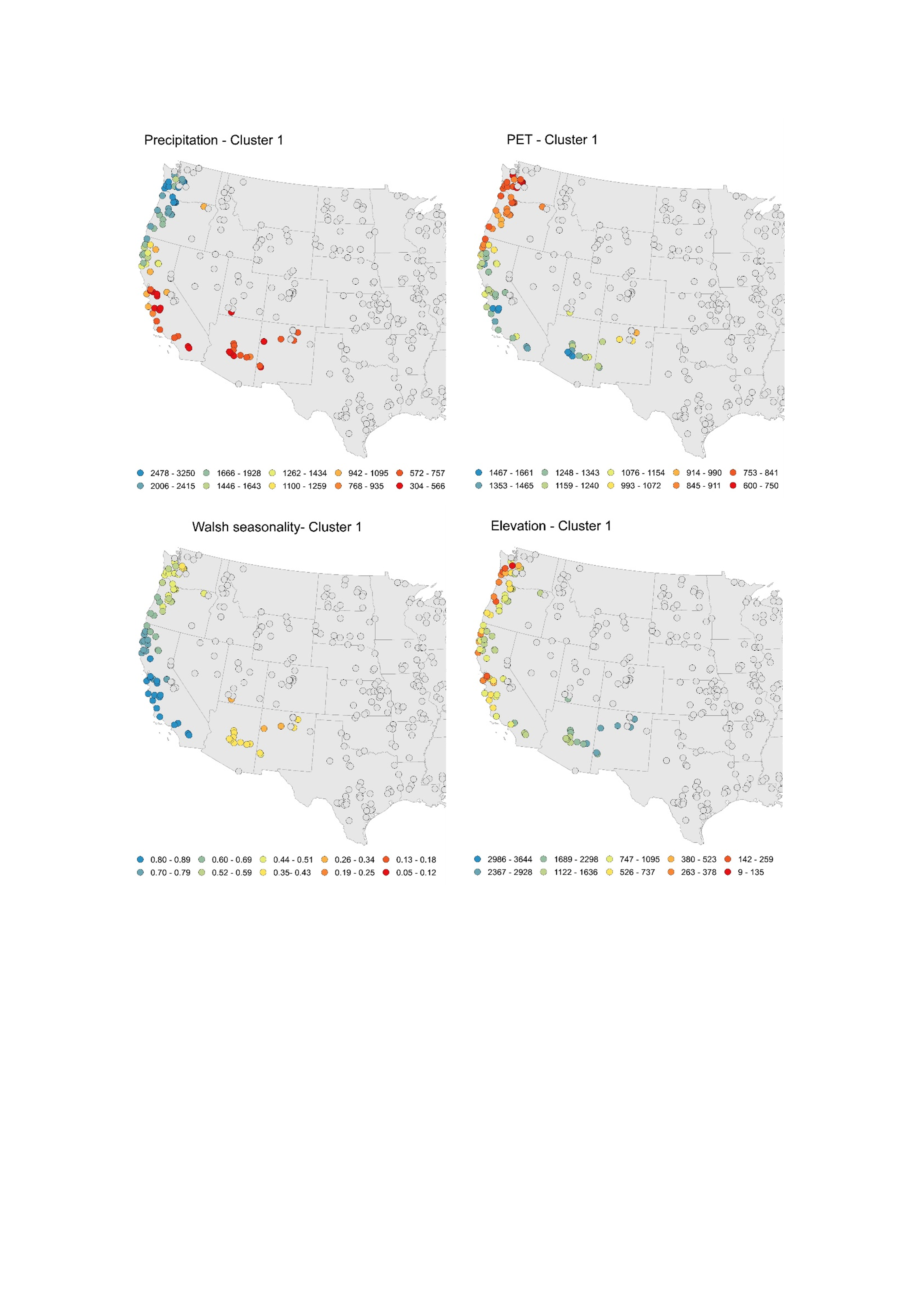

Supplement: Supplementary file 4 — Data S4 Supporting Information [file HYP-34-4-s004.tif]

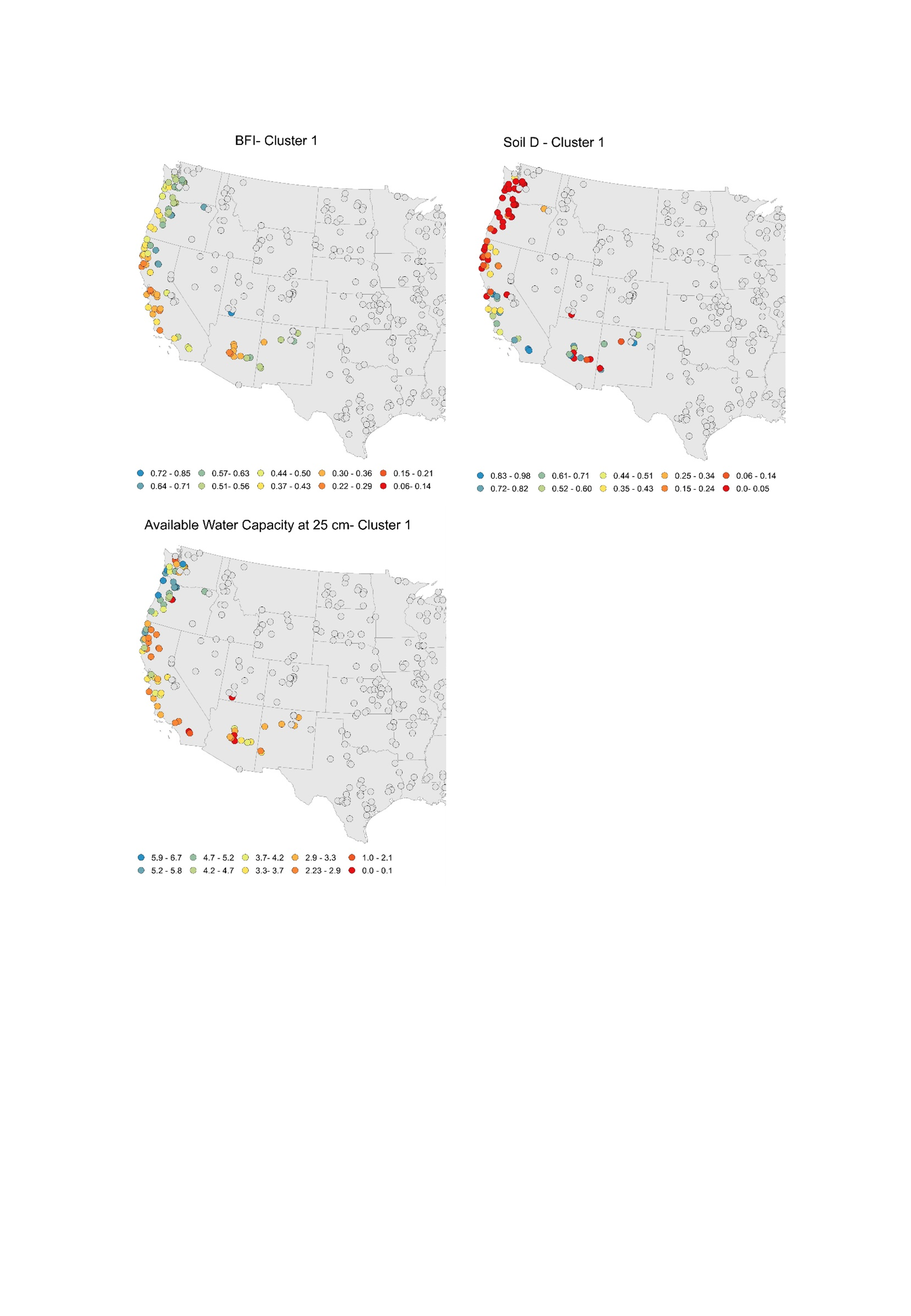

Supplement: Supplementary file 5 — Data S5 Supporting Information [file HYP-34-4-s005.tif]

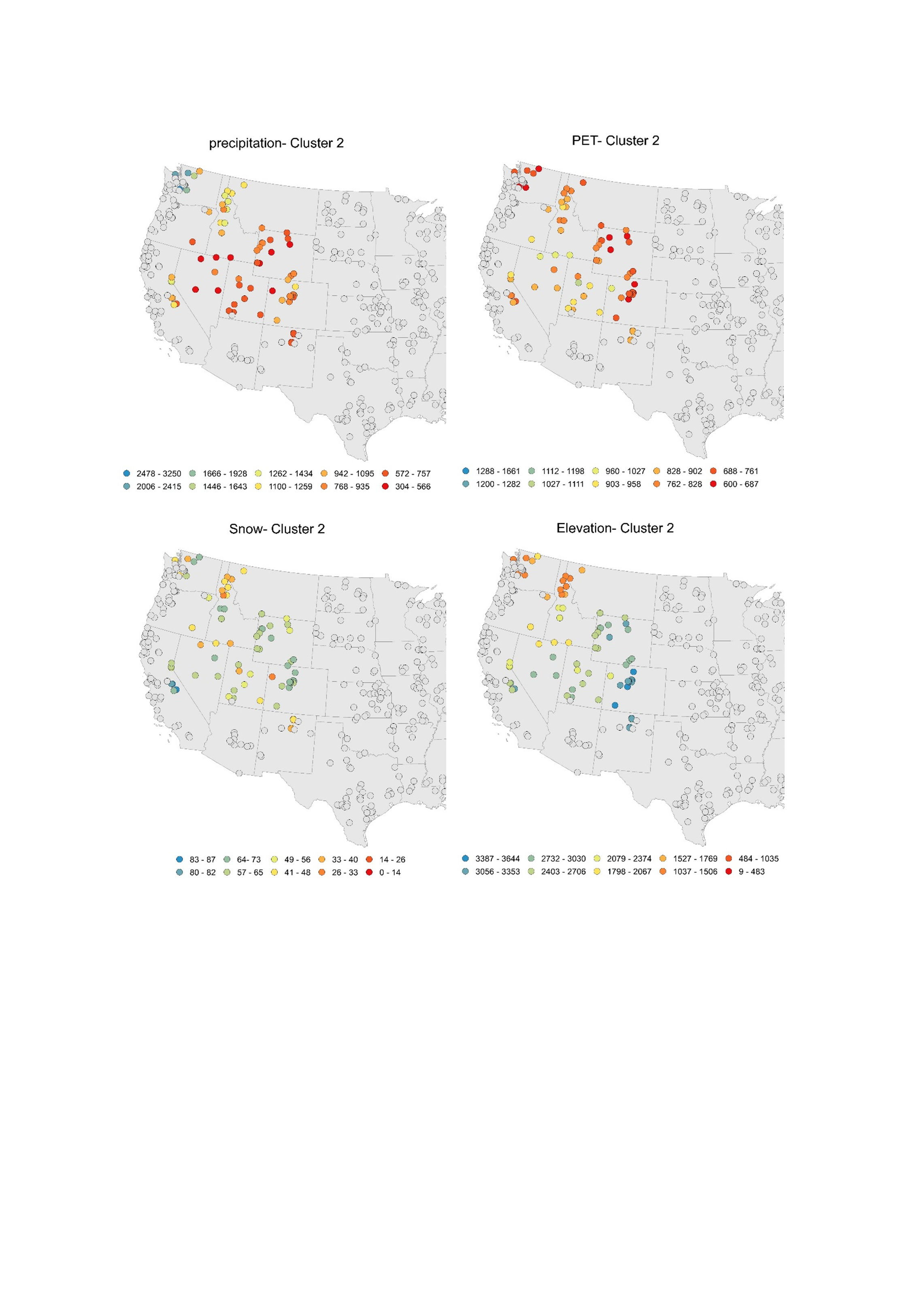

Supplement: Supplementary file 6 — Data S6 Supporting Information [file HYP-34-4-s006.tif]

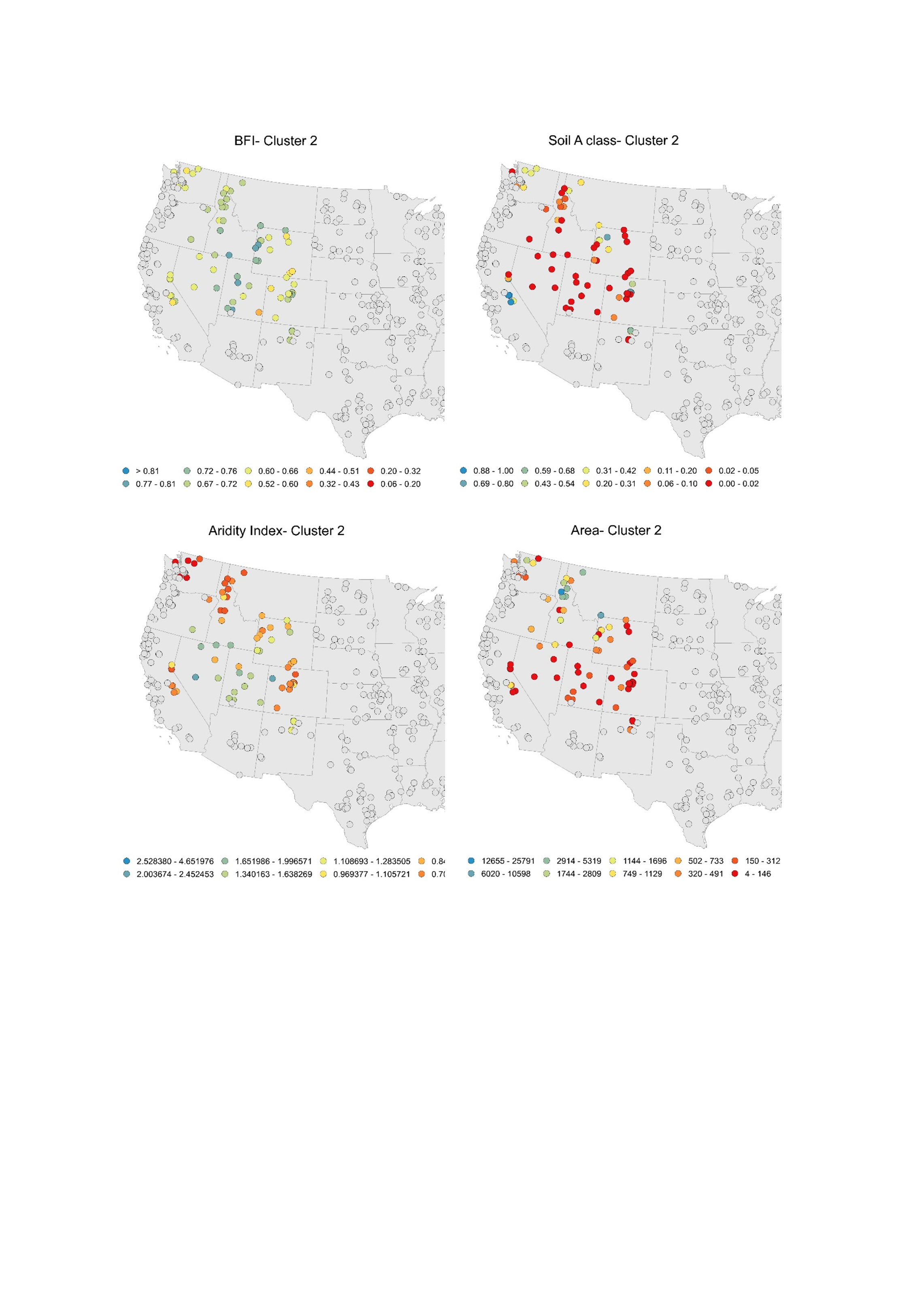

Supplement: Supplementary file 7 — Data S7 Supporting Information [file HYP-34-4-s007.tif]

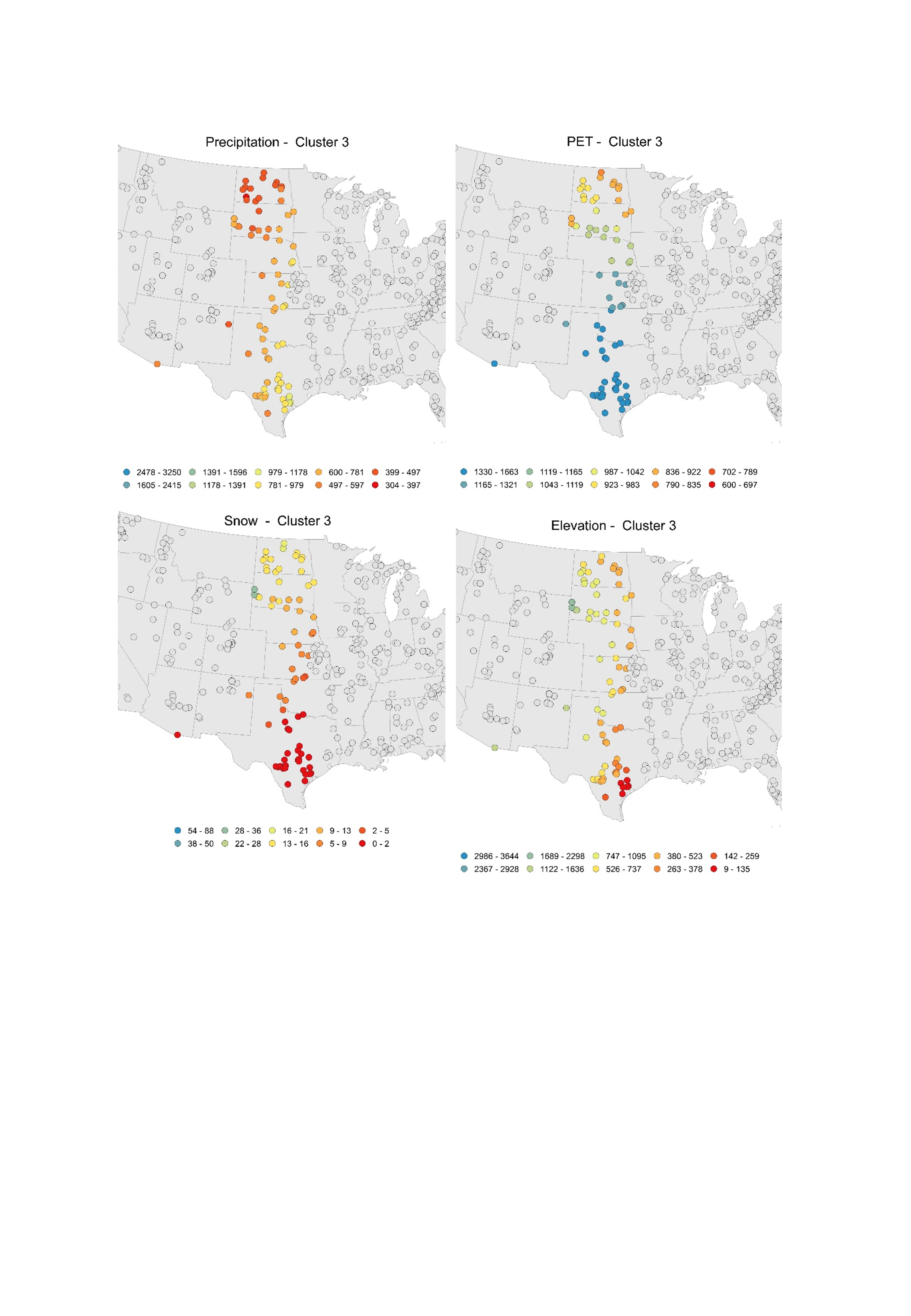

Supplement: Supplementary file 8 — Data S8 Supporting Information [file HYP-34-4-s008.tif]

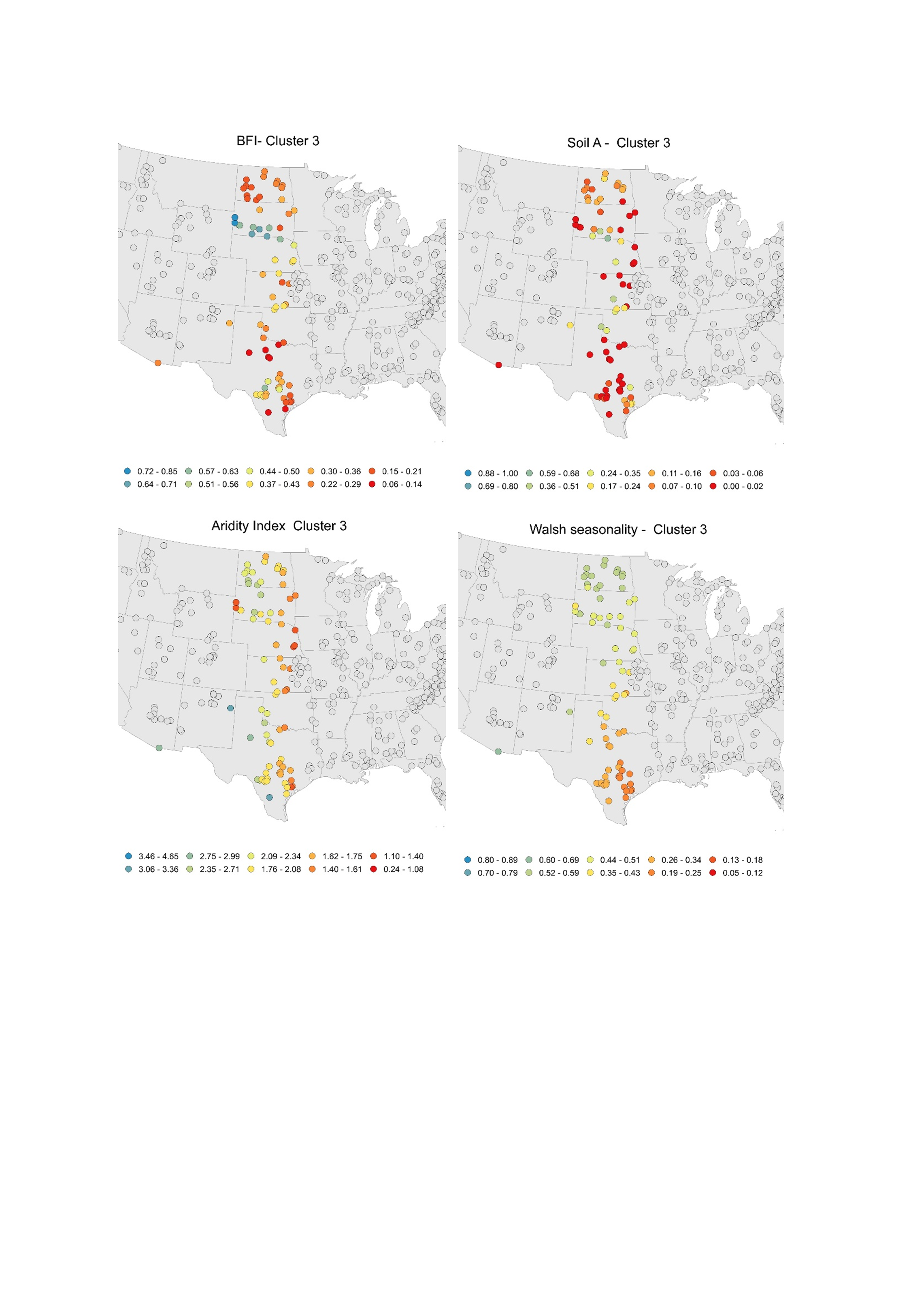

Supplement: Supplementary file 9 — Data S9 Supporting Information [file HYP-34-4-s009.tif]

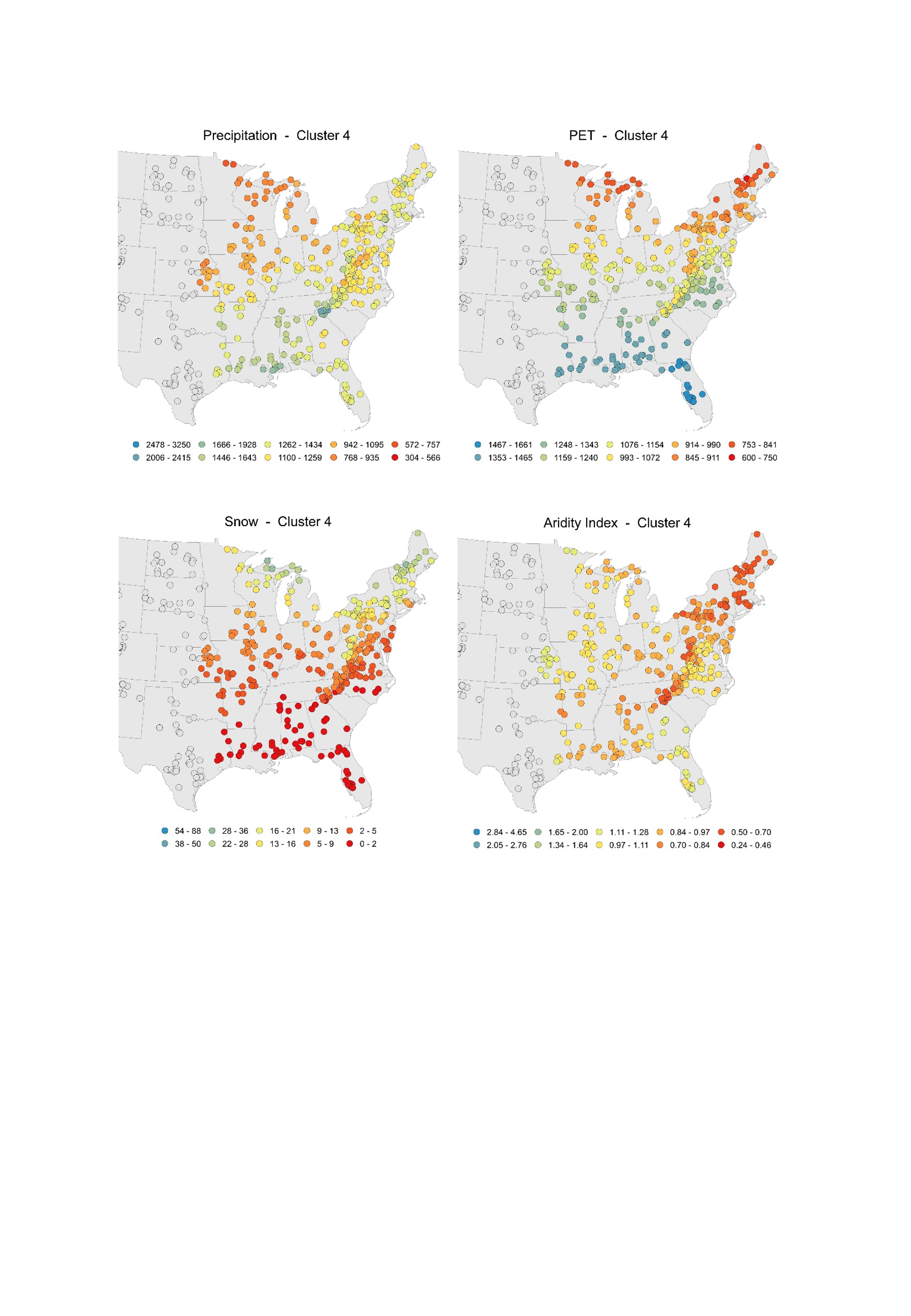

Supplement: Supplementary file 10 — Data S10 Supporting Information [file HYP-34-4-s010.tif]

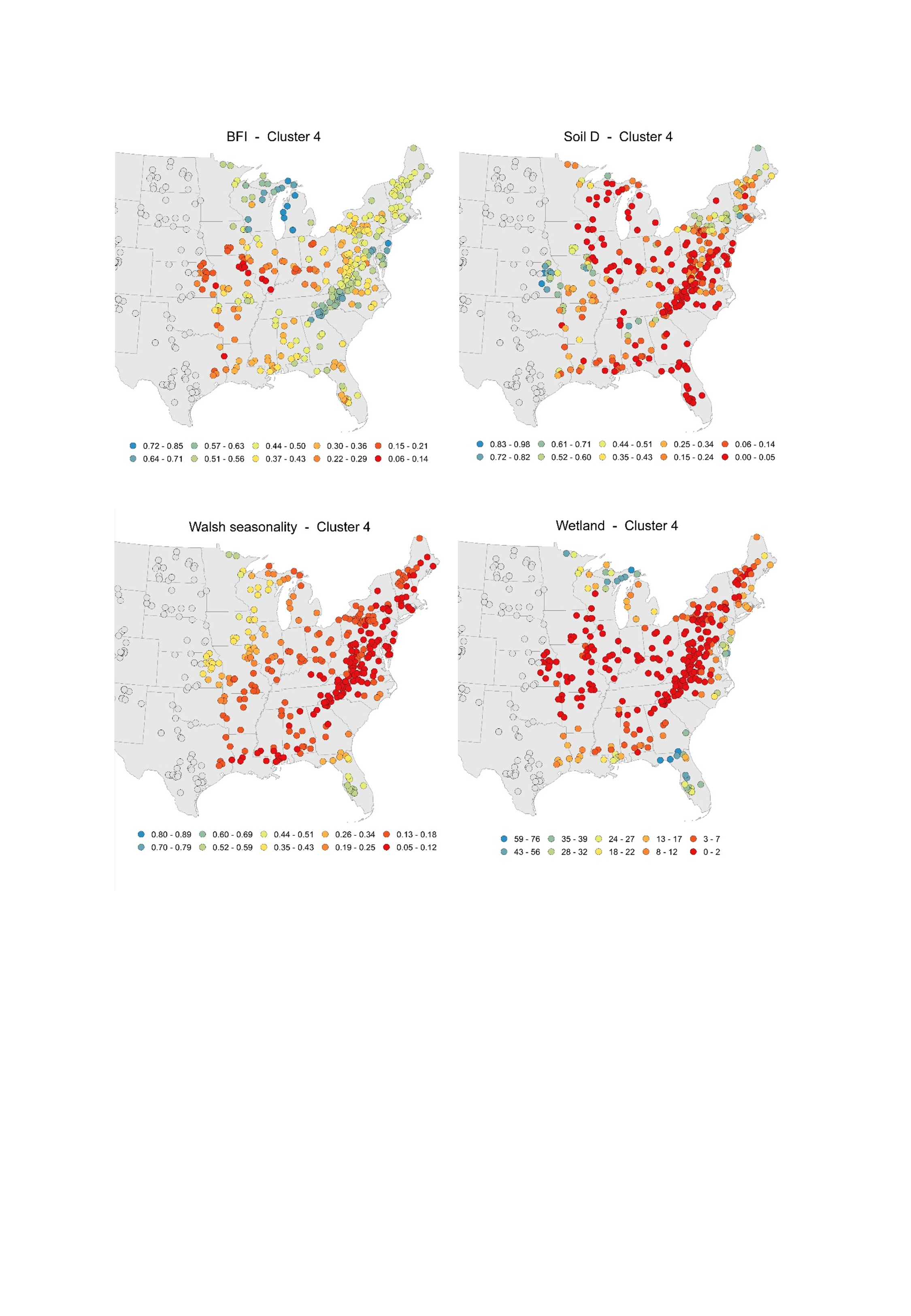

Supplement: Supplementary file 11 — Data S11 Supporting Information [file HYP-34-4-s011.tif]

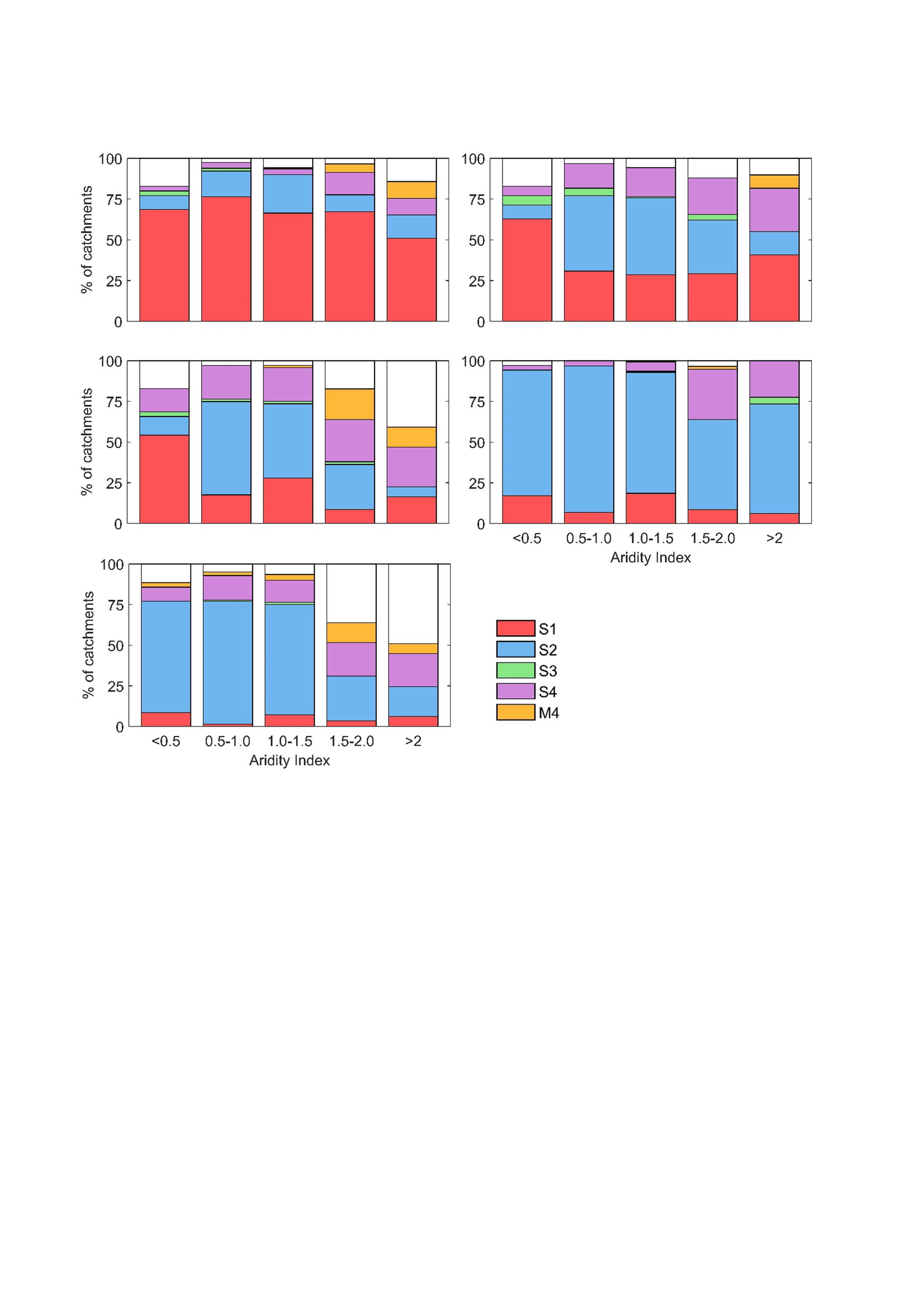

Supplement: Supplementary file 12 — Data S12 Supporting Information [file HYP-34-4-s012.tif]

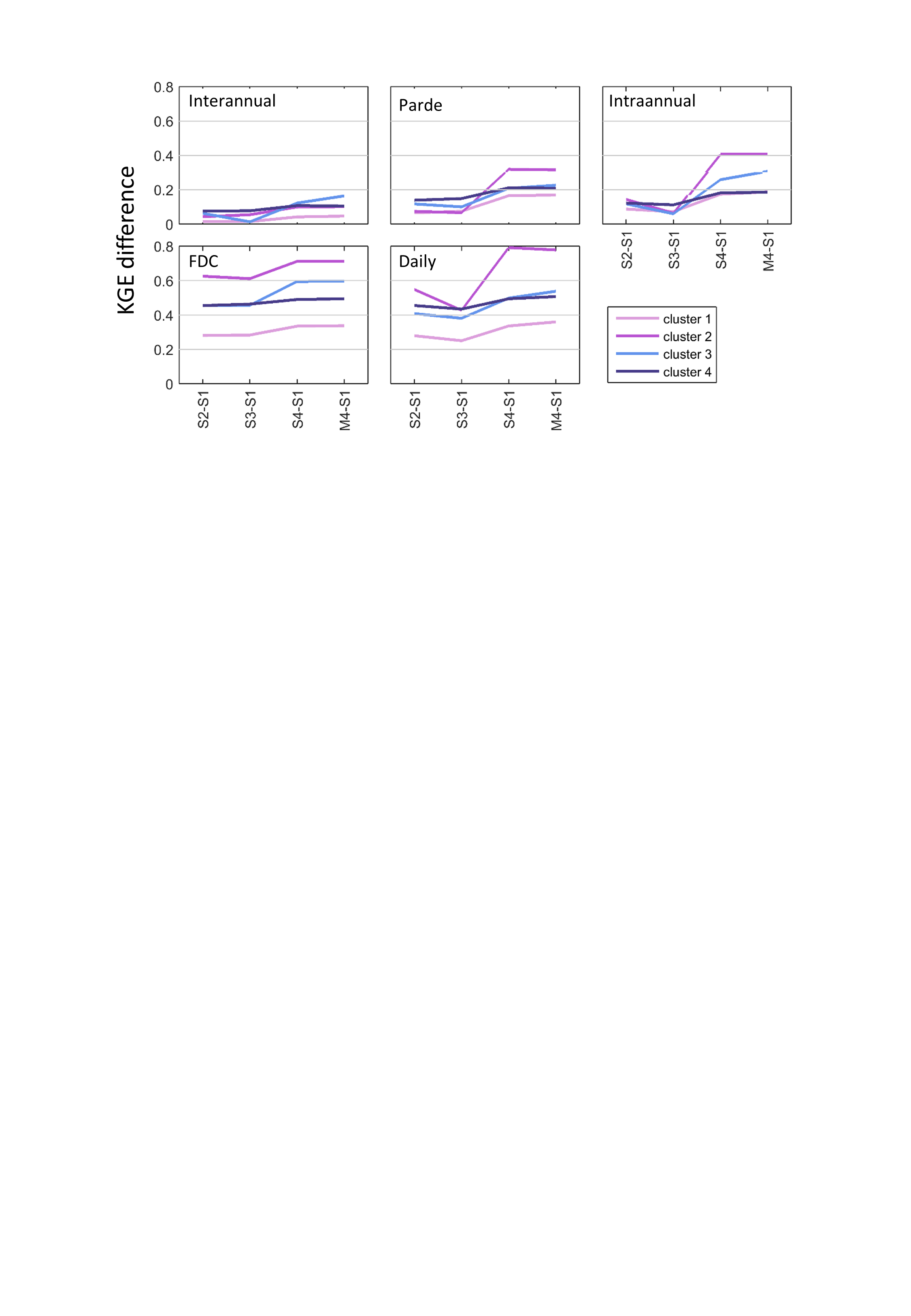

Supplement: Supplementary file 13 — Data S13 Supporting Information [file HYP-34-4-s013.tif]

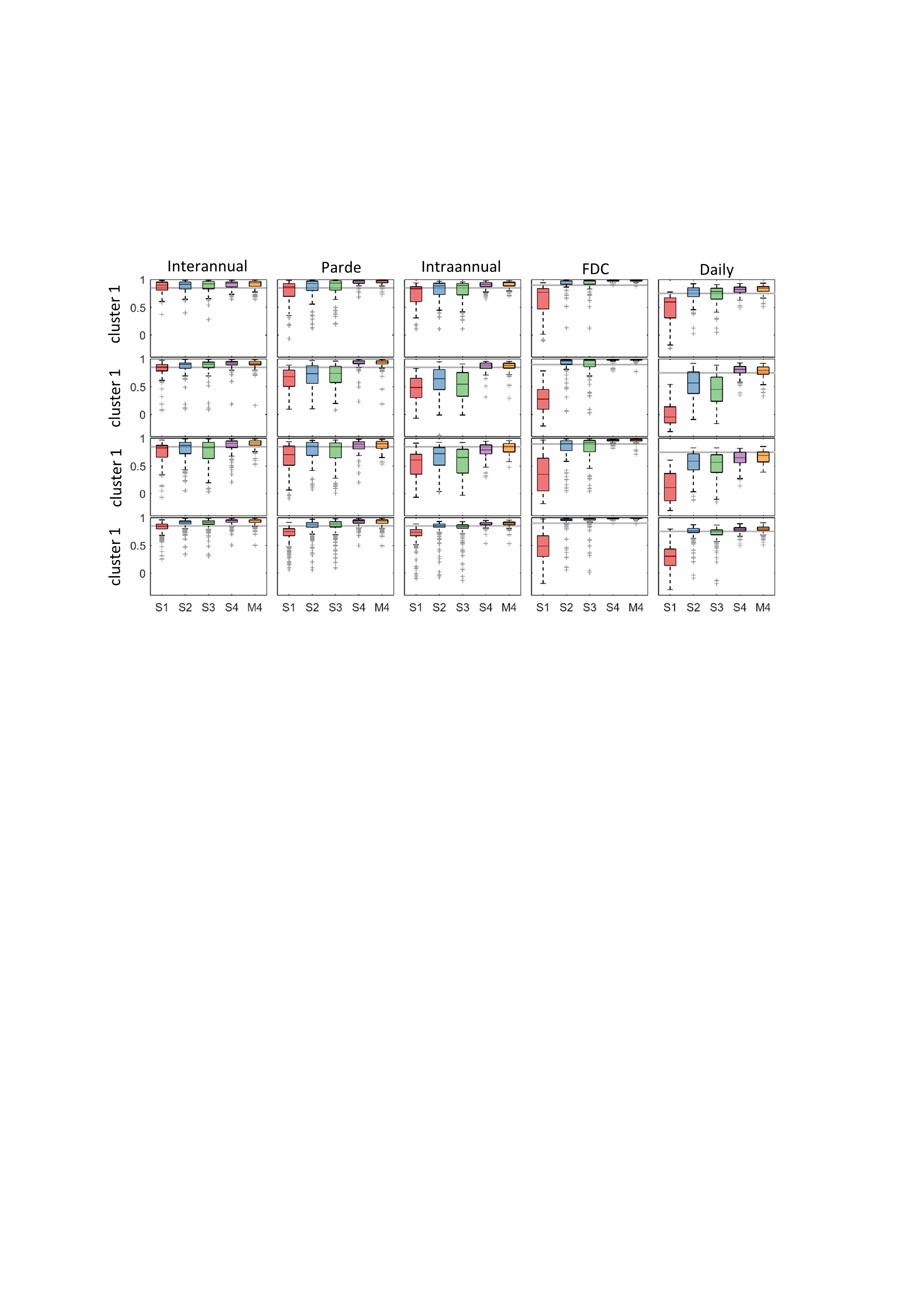

Supplement: Supplementary file 14 — Data S14 Supporting Information [file HYP-34-4-s014.tif]

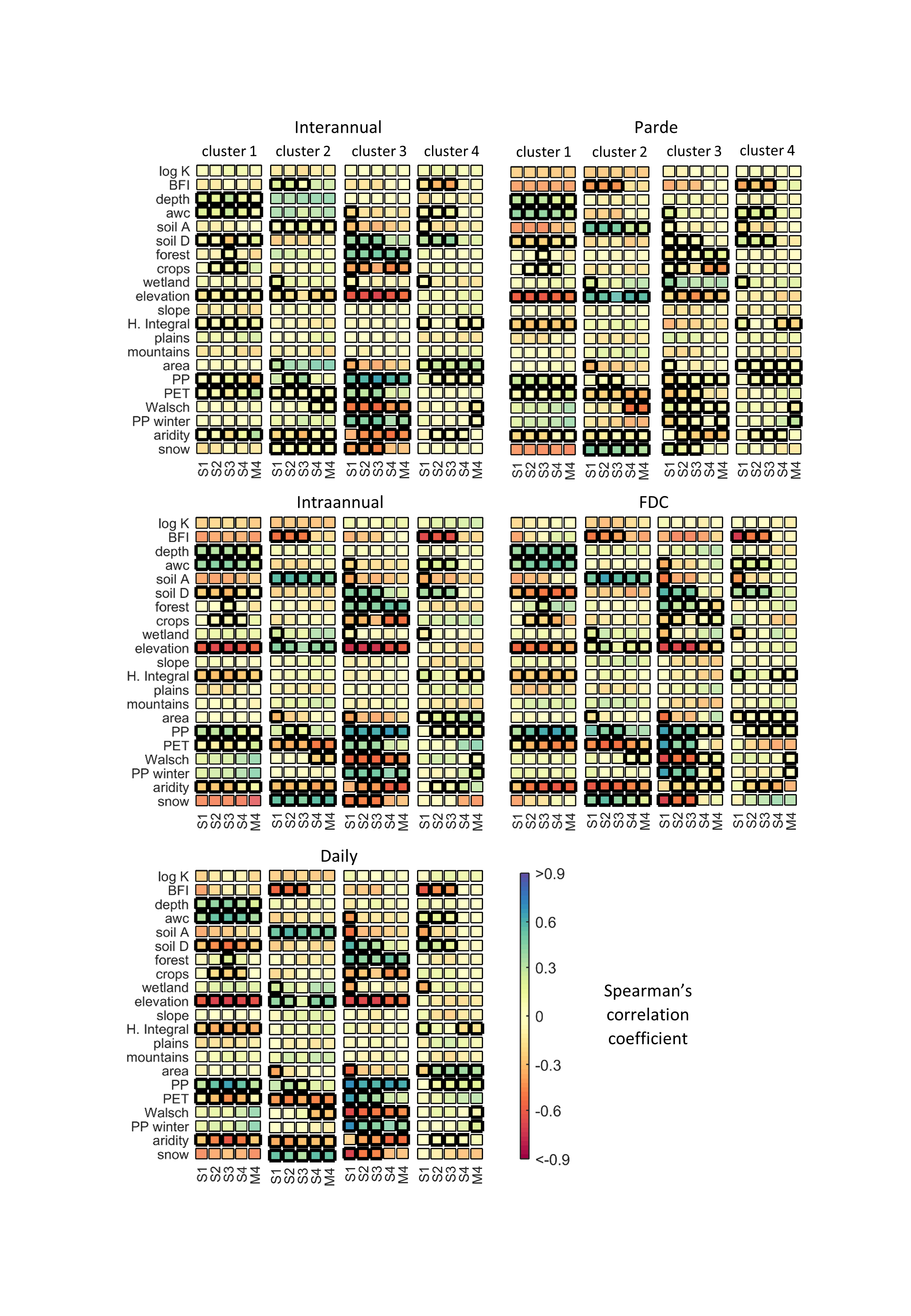

Supplement: Supplementary file 15 — Data S15 Supporting Information [file HYP-34-4-s015.tif]
