# Supplementary material for: Identification of factors influencing hydrologic model performance using a top‐down approach in a large number of U.S. catchments
Source: Hydrol Process. 2019 Nov 5;34(1):4–20. doi: 10.1002/hyp.13566 (PMC6973287; doi:10.1002/hyp.13566)
Supplement: Supplementary file 16 — Data S16 Supporting Information [file HYP-34-4-s016.docx]

**Model equations**

The model structures are taken from Bai et al. (2009), which in turn based them on the single bucket descriptions from Atkinson et al. (2002) and Farmer et al. (2003) and the multi bucket formulations from Son and Sivapalan (2007). Since we made some changes to the way some processes are calculated, we present the equations used in our study.

**Snow melt**

A snow melt and accumulation module is added to all models. It takes the precipitation and temperature time series as input and returns a value for snowmelt and liquid precipitation at each time step. This module was implemented for 100-m elevation bands, accounting in this way for the temperature variation with elevation, which was modelled with a lapse rate of -0.0064 °Cm^-1^.

(1) Estimating the temperature of each elevation band and allocating current precipitation to a liquid precipitation variable (used as input to the evapotranspiration modules) or to a snow accumulation variable

$$T_{k}=T-(el_{c}-el_{k}) lapse\_rate$$

$$P_{k}=\left\{\begin{matrix} PA_{k}, T_{k}\geq tt \\ 0, T_{k}<tt \end{matrix}\right.$$

$$AS_{k}=\left\{\begin{matrix} 0, T_{k}\geq tt \\ PA_{k}, T_{k}<tt \end{matrix}\right.$$

(2) Calculation of snow melt and update of snow accumulation state variable

$$SS_{k}=SS_{k \text{t-1}}$$

$$M_{p}=\max( 0,(T_{k}-tt) dd )$$

$$M_{k}=\left\{\begin{matrix} SS_{k}, M_{p}\geq SS_{k} \\ M_{p} M_{p}<SS_{k} \end{matrix}\right.$$

$$SS_{k}=\left\{\begin{matrix} AS_{k} M_{k}\geq SS_{k} \\ SS_{k}-M_{k}+AS_{k} M_{k}<SS_{k} \end{matrix}\right.$$

(3) Estimating rainfall and melt input for the whole catchment

$$P_{t}=sum (P_{k})$$

$$M=sum (M_{k})$$

**Model S1**

Model S1 consists of a single soil bucket, which produces discharge only due to saturation excess.

(1) Threshold parameter and threshold storage

$$f_{c}=\frac{(\theta_{fc}-\theta_{wp})}{(\varphi-\theta_{wp})}$$

$$S_{fc}=S_{b}f_{c}$$

(2) Evapotranspiration is calculated as the sum of interception, transpiration from soil covered by deep rooted vegetation and evaporation from bare soil

$$E_{i}=\alpha_{ei}P_{t}$$

$$S_{t}=\min( S_{b},S_{t-1}+P_{t}+M-E_{i})$$

$$E_{v}=\left\{\begin{matrix} NE_{p}, St\geq S_{fc} \\ NE_{p}\frac{S_{t}}{S_{fc}}, St<S_{fc} \end{matrix}\right.$$

$$E_{bs}=(1-N)\frac{S_{t}}{S_{b}}E_{p}$$

$$E=E_{i}+E_{v}+E_{bs}$$

(3) Calculation of discharge and update of current soil water level

$$S_{t}=S_{t-1}+P+M-E$$

$$Q_{se}=\left\{\begin{matrix} S_{t}-S_{b}, S_{t}\geq S_{b} \\ 0, S_{t}-S_{b} \end{matrix}\right.$$

$$Q=Q_{se}$$

$$S_{t}=S_{t}-Q_{se}$$

**Model S2**

Model S2 consists also of a single soil bucket, but it produces discharge from saturated flow besides saturation excess.

Steps (1) and (2) were already described for model S1.

(3) Calculation of discharge and update of current soil water level

$$S_{t}=S_{t-1}+P_{t}+M-E$$

$$Q_{se}=\left\{\begin{matrix} S_{t}-S_{b}, S_{t}\geq S_{b} \\ 0, S_{t}<S_{b} \end{matrix}\right.$$

$$S_{t}=S_{t}-Q_{se}$$

$$Q_{ss}=\left\{\begin{matrix} \alpha_{ss}(S_{t}-S_{fc}), S_{t}\geq S_{fc} \\ 0, S_{t}<S_{fc} \end{matrix}\right.$$

$$S_{t}=S_{t}-Q_{ss}$$

$$Q=Q_{se}+Q_{ss}$$

**Model S3**

Model S3 consists also of single soil bucket, which has a saturated and an unsaturated zone. Discharge occurs due to subsurface flow from the saturated zone as well as by saturation excess.

(1) Threshold parameter and threshold storage in the unsaturated zone

$$f_{c}=\frac{(\theta_{fc}-\theta_{wp})}{(\varphi-\theta_{wp})}$$

$$S_{ufc}=(S_{b}-S_{sat t-1})f_{c}$$

(2) Evapotranspiration is calculated as the sum of interception and transpiration from soil covered by deep rooted vegetation and evaporation from bare soil. Evaporation and transpiration occur in both, the saturated and unsaturated zones.

$$S_{us}=S_{t-1}-S_{sat t-1}$$

$$S'_{us}=S_{us}+P_{t}+M-E_{i}$$

$$S{}_{t}=\min(S_{b},S'_{us}+S_{sat t-1})$$

$$S_{sat}=\min( S_{b},S_{sat t-1}+r_{s})$$

$$S_{us}=S_{t}-S_{sat}$$

$$S_{usfc}=f_{c}(S_{b}-S_{sat})$$

$$E_{i}=\alpha_{ei}P_{t}$$

$$E_{v sat}=\frac{S_{sat}}{S_{t}}\frac{S_{t}}{S_{b}}NE_{p}$$

$$E_{bs sat}=\frac{S_{sat}}{S_{t}}\frac{S_{t}}{S_{b}}(1-N)E_{p}$$

$$E_{bs us}=\frac{S_{us}}{S_{t}}(1-N)\frac{S_{us}}{S_{b}-S_{sat}}\frac{S_{t}}{S_{b}}E_{p}$$

$$E_{v us}=\left\{\begin{matrix} \frac{S_{us}}{S_{t}}\frac{S_{t}}{S_{b}}NE_{p}, S_{us}>S_{usfc} \\ 0, S_{us}=0 \\ \frac{S_{us}}{S_{t}}N\frac{S_{us}}{S_{usfc}}\frac{S_{t}}{S_{b}}E_{p}, S_{us}<S_{usfc} \end{matrix}\right.$$

$$E_{bs}=E_{bs us}+E_{bs sat}$$

$$E_{v}=E_{v us}+E_{v sat}$$

$$E=E_{i}+E_{v}+E_{bs}$$

(3) Calculation of discharge and update of current soil water level

$$S_{t}=S_{t-1}+P_{t}+M-E$$

$$Q_{se}=\left\{\begin{matrix} S_{t}-S_{b}, S_{t}\geq S_{b} \\ 0, S_{t}<S_{b} \end{matrix}\right.$$

$$Q_{ss}=\alpha_{ss}S_{sat}$$

$$Q=Q_{se}+Q_{ss}$$

$$S_{sat}=S_{sat}-Q_{ss}$$

$$S_{t}=S_{t}-Q$$

**Model S4**

Model S4 has the same structure as model S3 for the soil storage, but it has additionally a deep storage which is recharged by the saturated zone of the soil bucket. There is no evapotranspiration from this store which thus loses water only through discharge.

Steps (1) and (2) are the same as for model S3.

(3) Recharge to deep store and discharge from it

$$Q_{bf}=\alpha_{bf}S_{deep}$$

$$r_{g}=k_{d}S_{sat}$$

$$S_{deep}=S_{deep}-Q_{bf}+r_{g}$$

(4) Calculation of discharge and update of current soil water level

$$S_{t}=S_{t-1}+P_{t}+M-E$$

$$Q_{se}=\left\{\begin{matrix} S_{t}-S_{b}, S_{t}\geq S_{b} \\ 0, S_{t}<S_{b} \end{matrix}\right.$$

$$S_{sat}=S_{sat}-r_{g}$$

$$Q_{ss}=\alpha_{ss}S_{sat}$$

$$S_{sat}=S_{sat}-Q_{ss}$$

$$Q=Q_{se}+Q_{ss}+Q_{bf}$$

$$S_{t}=S_{t}-Q_{se}-Q_{ss}-r_{g}$$

**Multi-soil-bucket models**

Models M1 – M4 have the same structure as the corresponding models S1 – S4. They do have, however, 10 soil storages instead of a single soil storage for representing the spatial variability of soil depth in the catchments.

(1) Calculation of the size of each storage

$$S_{\max}=(1+b)S_{b}$$

$$F=[0.05 0.15 0.25 0.35 0.45 0.55 0.65 0.75 0.85 0.95]$$

$$S_{b\_f}=S_{\max} [1-(1-F)^{1/b}]$$

Evapotranspiration and discharge are calculated for each storage independently following the descriptions presented for models S1-S4. The total discharge at each time step is then calculated as the sum of the discharge from all storages.

**NOTATION**

| $\alpha_{bf}$ | baseflow recession coefficient (d^-1^) |
| --- | --- |
| $\alpha_{ss}$ | saturation flow recession coefficient (d^-1^) |
| $\alpha_{ei}$ | interception coefficient (dimensionless) |
| $\varphi$ | total soil porosity (%) |
| $\theta_{fc}$ | field capacity (%) |
| $\theta_{wp}$ | permanent wilting point (%) |
| $b$ | shape parameter describing the spatial distribution of soil moisture in the multi-bucket models (dimensionless) |
| $dd$ | day degree factor for snow melt (mm°C^-1^d^-1^) |
| $el_{c}$ | median catchment elevation (m) |
| $el_{k}$ | vector of median elevation of each elevation band (m) |
| $f_{c}$ | threshold storage parameter (dimensionless, 0 <$f_{c}$<1) |
| $k_{d}$ | recharge coefficient from soil storage to deep storage (d^-1^) |
| $r_{s}$ | recharge from unsaturated to saturated zone in soil storage (mmd^-1^) |
| $r_{p}$ | recharge from soil storage to deep storage (mmd^-1^) |
| $tt$ | threshold temperature for snow accumulation and melt (°C) |
| $A_{s}$ | vector of fraction of catchment area in each elevation band (dimensionless) |
| $AS_{k}$ | vector of additional snow accumulation in each elevation band at current time step (mm) |
| $E$ | actual evapotranspiration (mmd^-1^) |
| $E_{p}$ | potential evapotranspiration (mmd^-1^) |
| $E_{i}$ | evaporation loss due to interception (mmd^-1^) |
| $E_{bs}$ | evaporation from bare soil (mmd^-1^) |
| $E_{bs sat}$ | evaporation from saturated bare soil (mmd^-1^) |
| $E_{bs us}$ | evaporation from unsaturated bare soil (mmd^-1^) |
| $E_{v}$ | transpiration from soil covered by deep rooted vegetation (mmd^-1^) |
| $E_{v sat}$ | transpiration from saturated soil (mmd^-1^) |
| $E_{v us}$ | transpiration from unsaturated soil (mmd^-1^) |
| $k$ | index identifying each elevation band |
| $F$ | vector describing the normalised cumulative spatial distribution of the buckets in the multi-bucket models (dimensionless) |
| $M$ | total snow melt in the catchment (mmd^-1^) |
| $M_{k}$ | snow melt in each elevation band (mmd^-1^) |
| $M_{p}$ | potential snow melt (mmd^-1^) |
| $N$ | fraction of catchment covered by deep rooted vegetation (dimensionless) |
| $P$ | precipitation (mmd^-1^) |
| $P_{eb}$ | vector with liquid precipitation input in each elevation band (mmd^-1^) |
| $P_{t}$ | total liquid precipitation input into the catchment (mmd^-1^) |
| $Q$ | total discharge (mmd^-1^) |
| $Q_{bf}$ | discharge from deep storage (mmd^-1^) |
| $Q_{se}$ | discharge from saturation excess (mmd^-1^) |
| $Q_{ss}$ | discharge from subsurface storage (mmd^-1^) |
| $S_{b}$ | size of soil storage in single bucket models (mm) |
| $S_{fc}$ | threshold storage (mm) |
| $S_{t}$ | water level in soil storage at current time step (mm) |
| $S_{t-1}$ | water level in soil storage at previous time step (mm) |
| $S_{sat}$ | water level in saturated zone at current time step (mm) |
| $S_{sat t-1}$ | water level in saturated zone at previous time step (mm) |
| $S_{us}$ | water level in unsaturated zone at current time step (mm) |
| $S_{usfc}$ | field capacity of unsaturated zone at current time step (mm) |
| $S_{deep}$ | water level in deep storage at current time step (mm) |
| $S_{\max}$ | maximum soil water storage in the catchment for multi-bucket models (mm) |
| $S_{b\_f}$ | vector of size of soil water storage for each bucket in multi-bucket models (mm) |
| $SS_{k}$ | vector of water stored as snow in each elevation band at the current time step (mm) |
| $SS_{k t-1}$ | vector of water stored as snow in each elevation band at previous time step (mm) |
| $T$ | mean air temperature at current time step (°C) |
| $T_{k}$ | vector of temperature in each elevation band at current time step (°C) |

**PARAMETER RANGES**

| parameter | description | range | unit |
| --- | --- | --- | --- |
| $N$ | fraction of catchment with deep rooted vegetation | 0 - 1 | - |
| $\alpha_{ei}$ | interception coefficient | 0 - 0.49 | - |
| $dd$ | day degree factor for snow melt | 1 - 12 | mm°C^-1^d^-1^ |
| $tt$ | threshold temperature for snow accumulation and melt | -5 - 5 | °C |
| $S_{b}$ | size of soil bucket | 0 - 1200 | mm |
| $\theta_{fc}$ | field capacity | 0 - 100 | % |
| $\alpha_{ss}$ | recession coefficient for subsurface flow | 0.05 - 0.5 | d^-1^ |
| $\alpha_{bf}$ | recession coefficient for deep storage | 0.001 - 0.05 | d^-1^ |
| $b$ | shape parameter for spatial soil water distribution | 0.1 - 2.5 | - |
| $k_{d}$ | percolation coefficient describing recharge from soil storage to the deep storage | 0 - 0.5 | d^-1^ |

**PARAMETERS INCLUDED IN EACH MODEL**

|  | | **Parameters** | | | | | | | | | |
| --- | --- | --- | --- | --- | --- | --- | --- | --- | --- | --- | --- |
|  |  | **M** | **α_ei_** | **dd** | **tt** | **Sb** | **fc** | **α_ss_** | **α_gw_** | **K_d_** | **b** |
| **Models** | **S1** | x | x | x | x | x | x |  |  |  |  |
|  | **S2** | x | x | x | x | x | x | x |  |  |  |
|  | **S3** | x | x | x | x | x | x | x |  |  |  |
|  | **S4** | x | x | x | x | x | x | x | x | x |  |
|  | **M1** | x | x | x | x | x | x |  |  |  | x |
|  | **M2** | x | x | x | x | x | x | x |  |  | x |
|  | **M3** | x | x | x | x | x | x | x |  |  | x |
|  | **M4** | x | x | x | x | x | x | x | x | x | x |

**A1: Catchment characterization**

The following plots show the distribution of different catchment properties across all considered catchments (Figure A1).

**A2: Identification of catchments with problems**

The presence of catchments with runoff coefficients larger than one motivated the implementation of a screening step for eliminating catchments with problems in the input data. A conservative approach was used. It identifies the catchments with problems in reproducing the water balance. There might still be catchments with a satisfactory overall water balance but with other types of input errors which were not identified by this methodology.

Figure A2 shows the distribution of the normalised absolute bias for all catchments. The minimum bias achieved with any model and MC run was assigned to each catchment

The arrow indicates the bias threshold separating the catchments with acceptable input data (yellow) from the catchments that were eliminated (blue).

We eliminated 13 catchments all of which had a bias > 0.0001.

**A3 and A4: Spatial distribution of catchment predictors in cluster 1**

Figures A3 and A4 show the distribution of the predictors in cluster 1.

**A5 and A6: Spatial distribution of catchment predictors in cluster 2**

Figures A5 and A6 show the distribution of the predictors in cluster 2.

**A7 and A8: Spatial distribution of catchment predictors in cluster 3**

Figures A7 and A8 show the distribution of the predictors in cluster 3.

**A9 and A10: Spatial distribution of catchment predictors in cluster 4**

Figures A9 and A10 show the distribution of the predictors in cluster 4.

**T1: Results for model calibration**

Table A1 shows the performance of the models in the calibration period.

**A11: Relationship between model performance and the aridity index**

Figure A11 is analogous to Figure 2 in the text, but shows the results for the calibration period.

**A12:**

Figure A12 is analogous to Figure 5 in the text, but shows the results for the calibration period.

**A13:**

Figure A13 is analogous to Figure 6 in the text, but shows the results for the calibration period.

**A14:**

Figure A14 is analogous to Figure 7 in the text, but shows the results for the calibration period.
